# Today, Tomorrow, and Overmorrow: The Acquisition of Deictic Temporal Terms in English and German

**DOI:** 10.1162/OPMI.a.254

**Published:** 2025-10-29

**Authors:** Katherine Steele, Anna Bánki, Gabriela Markova, Stefanie Hoehl, Katharine A. Tillman

**Affiliations:** Department of Psychology, University of Texas at Austin, Austin, TX, USA; Faculty of Psychology, University of Vienna, Vienna, Austria

**Keywords:** word learning, cross-linguistic comparison, time, language acquisition, abstract concepts, timeline, calendar, yesterday, tomorrow

## Abstract

English and German both have single words for *yesterday* and *tomorrow*, but German also includes the words *vorgestern* (“the day before yesterday”) and *übermorgen* (“the day after tomorrow”). This study investigates how these differences in time-word sets influence children’s learning of temporal language. In two tasks, English- and German-speaking children ages 3 to 7 (*N* = 304) and adult controls (*N* = 75), marked the locations of temporal terms on a continuous timeline and a discontinuous calendar template. We assessed knowledge of three distinct facets of time-meaning (past/future status, sequential ordering, and temporal remoteness) and precise meanings for temporal terms. Our results show that German-speaking children were more likely to demonstrate precise understanding of items lexicalized only in German (e.g., *übermorgen*/*day-after-tomorrow)* as well as basic time words lexicalized in both languages (e.g., *gestern*/*yesterday*). The German advantage was primarily driven by children’s better understanding of these words’ temporal remoteness (i.e., distance from the present), while there were no language-group differences in children’s understanding of past/future status. These findings suggest that children acquire time-word meanings gradually, using different linguistic cues for different facets of meaning, and that having a more extensive time-word lexicon may help constrain German-speaking children’s early understanding of temporal concepts like *yesterday* and *tomorrow*.

## INTRODUCTION

How do young children experience, think about, and encode time in language? While time perception (e.g., discrimination of temporal durations) emerges in infancy (e.g., Gava et al., 2012), fully adult-like temporal language use develops much later. English-speaking children begin producing time-related words between ages 2 and 3 (e.g., Ames, 1946; Busby & Suddendorf, 2005; Grant & Suddendorf, 2011; Suddendorf, 2010), and 4-year-olds use both duration words (e.g., *minute*) and deictic time words (e.g., *yesterday*) in appropriate contexts (e.g., Shatz et al., 2010; Tillman & Barner, 2015; Tillman et al., 2017). However, children acquire precise meanings for temporal terms gradually, with some studies suggesting that they do not reach full, adult-like accuracy until between ages 7 and 8 (Grant & Suddendorf, 2011; Harner, 1975; McCormack, 2015; Tillman et al., 2017). This study examines the acquisition of deictic temporal terms^1^ across languages, specifically comparing English and German. While both languages have similar tense structures and single words denoting “yesterday” and “tomorrow,” German contains additional words for “the day before yesterday” (*vorgestern*) and “the day after tomorrow” (*übermorgen*), equivalent to the now-defunct Old English words “ereyesterday” and “overmorrow.” Leveraging this difference in time-word lexicons, we investigate whether English- and German-speaking children aged 3 to 7 differ in their early acquisition of temporal terms, and, in particular, in their understanding of the basic terms like gestern/yesterday that are lexicalized in both languages. We also examine the theoretical implications of these differences for how time words, and other abstract terms, are learned.

Deictic time words encode temporal relationships between events and the present (e.g., *yesterday* vs. *tomorrow*). Their reference depends on context, posing a particular challenge for language learners (e.g., Tuesday’s *tomorrow* is different from Wednesday’s *tomorrow*). Young children produce words for more present times (e.g., *now*, *today*) earlier than those referring to more distal times (e.g., *tomorrow*, *yesterday*, *last week*, etc.; see Frank et al., 2017; Grant & Suddendorf, 2011). Importantly, the production of deictic time words precedes full comprehension of their meanings. Although many English-speaking 3-year-olds produce *yesterday* and *tomorrow*, only about 30% can successfully identify events occurring on those days (Busby & Suddendorf, 2005), likely reflecting limitations in comprehension or memory. Tillman et al. (2017) found that it takes English-speaking children four or more years after production begins to acquire the precise meanings of deictic time words. That is, although 4-year-old English-speakers understand that *yesterday* refers to a time in the past, understanding that it signifies “exactly one day before today” emerges only after age 7.

While multiple studies indicate that English-speaking children initially struggle to comprehend deictic time words (Busby & Suddendorf, 2005; Grant & Suddendorf, 2011; Tillman et al., 2017), there is considerable variability in the methods used to assess children’s knowledge and in researchers’ conclusions about the age at which children ultimately acquire that knowledge. For example, when asked to select a toy “from yesterday” or “for tomorrow”, 3-year-olds make fewer errors in response to *yesterday* (Harner, 1975), and 5-year-olds are still more accurate in answering questions about what happened *yesterday* relative to questions about what will happen *tomorrow* (Zhang & Hudson, 2018). Other studies demonstrate that children’s accuracy is impacted by a word’s remoteness, or relative distance from the present (e.g., Hudson & Mayhew, 2011; Tillman et al., 2017). For example, Hudson and Mayhew (2011) showed that 5- to 6-year-olds were less accurate at judging the relative temporal distances of terms referring to far distances (e.g., *two weeks ago*) than words referring to near distances (e.g., *tomorrow*) in relation to “today”. By age 7, however, children reliably understand both near and distant time words (Tillman et al., 2017).

Although this diversity in research methods has complicated the literature on English-speakers’ acquisition of deictic temporal terms somewhat, there is relatively little work examining deictic time-word acquisition in populations that speak languages other than English (but see Maheshwari & Barner, 2025, for a recent comparison of English and Hindi, and numerous classic studies by Weist examining acquisition of temporal language in Polish, e.g., Weist & Buczowska, 1987). As we discuss further below, languages vary substantially in how they express time, both syntactically (e.g., via grammatical tense and aspect) and semantically (e.g., in the sets of time words they contain). These cross-linguistic differences provide fertile ground for exploring how different linguistic cues and features influence children’s learning of particularly difficult, abstract terms.

One reason that acquiring adult-like meanings for deictic temporal terms may be challenging for children to acquire is because their meanings are multifaceted. In English, for example, understanding these terms requires integrating (1) *deictic status*, whether the event is in the past or future, (2) *temporal remoteness*, the precise distance of the event in relation to the present, and (3) *sequential order*, the temporal ordering of events in relation to one another. With age, children learn to integrate these three facets of meaning to acquire *precise meanings* consistent with an adult-like understanding (e.g., knowing that *tomorrow* is a reference to exactly one day in the future from *today*; Tillman et al., 2017). One study examining English-speaking children’s comprehension and acquisition of partial meanings for time words in terms of these three facets of meaning suggests that children systematically and independently acquire these facets of meaning over several years. To assess this, Tillman et al. (2017) asked 3- to 8-year-old English-speakers to indicate where deictic time words were located on timelines extending from the past (their infancy) to the future (their adulthood). By age 4, children could demonstrate knowledge of deictic status, accurately placing items to the left (past) vs. right (future) of ‘now.’ Knowledge of deictic status emerged in tandem with knowledge of sequential ordering, i.e., that *last week* is farther in the past than *yesterday*. In contrast, temporal remoteness developed much later, around age 7, as children learn that *yesterday* specifically refers to precisely one day from the present.

The findings reviewed above raise important questions about how children bootstrap the meanings of time words. Linguistic cues, such as grammatical tense, may support early learning of deictic status in English. For instance, tense markings in sentences like “I *cleaned* yesterday” highlight that *yesterday* refers to the past. Research shows that 2- and 3-year-olds can already use tense to distinguish between past, present, and future actions (Wagner, 2001). Similarly, English-speaking children may infer sequential order through cues like contrastive usage or order-of-mention. For example, in the sentence “*Last week* she took the train, but *yesterday* she walked”, contrast and temporal ordering indicate that *last week* is further back in time than *yesterday*. However, temporal remoteness lacks straightforward linguistic cues in English, potentially explaining its delayed acquisition.^2^

Investigating how time words are learned in languages with different tense structures and sets of time-words can reveal whether and how linguistic cues shape the trajectory of learning. For example, a recent study by Maheshwari and Barner (2025) examined the comprehension of “yesterday” and “tomorrow” in children learning English and Hindi. Hindi contains a single term (“kal”) which refers to times one day from the present, and its past vs future reference must therefore be derived from the grammatical tense of the sentence in which it is used. Nevertheless, Maheshwari and Barner (2025) found that Hindi-speakers showed greater comprehension of sentences containing this term than English-speakers did of sentences containing yesterday/tomorrow. Furthermore, English-speakers struggled when tense cues were removed from the prompts. Both findings are consistent with the hypothesis that grammatical tense plays a strong role in bootstrapping children’s understanding of the deictic status of “yesterday” and “tomorrow.”

Here, we also take a cross-linguistic approach, but we compare English and German. Although both of these languages encode deictic status using tense (“I painted yesterday”/“Ich habe gestern gemalt”) and both contain single words to refer to a time exactly one day in the future (*tomorrow*/*morgen*) and exactly one day in the past (*yesterday*/*gestern*), only German includes single, compound words for “the day before yesterday” (*vorgestern*) and “the day after tomorrow” (*übermorgen*). In contrast, English lacks these words, and must use whole phrases to designate these time points. This difference allows us to ask, first, whether German-speaking children show earlier acquisition of the terms that are lexicalized only in German, *vorgestern* and the *übermorgen*, compared to English-speaking children acquiring the equivalent phrases. Secondly, and more interestingly, we also ask whether this difference in the overall structure of the time-word lexicons in the two languages influences children’s learning of the basic terms yesterday/gestern and tomorrow/morgen that are lexicalized in both languages. Specifically, we examine differences in children’s knowledge of the precise (adult-like) meaning of each term, as well as their knowledge of individual facets of their meanings—deictic status, sequential order, and temporal remoteness—which could be supported by different linguistic cues.

Learning a single word like *vorgestern* might seem at first blush to be easier than acquiring a complex phrase (“the day before yesterday”), especially considering English-speakers’ known difficulties with comprehension of “before” and “after” (e.g., Amidon & Carey, 1972; Blything & Cain, 2016, 2019; Blything et al., 2015; Cain & Nash, 2011; Clark, 1971). However, there is relatively little prior work on German-speakers’ early comprehension of prefixes like *vor-* and *über-* (Mattes, 2019), and to our knowledge, no prior work investigating German-speakers’ acquisition of deictic time words specifically (see Williams et al., 2021 for preliminary findings using a subset of data from the present study). In principle, early challenges in understanding the concept of “exactly two days in the past” could render comprehension of both types of expressions equally difficult. We therefore predicted that we would either find an advantage for German-speakers on the items that are lexicalized only in German, or, if the bottleneck on learning is conceptual, no difference between language groups on these items.

From a theoretical standpoint, however, an even more compelling question is whether access to a denser and more extensive temporal lexicon influences acquisition of the more basic terms that are lexicalized in both languages (e.g., *gestern*/*yesterday* and *morgen*/*tomorrow*). Prior work on English-speakers’ acquisition of both deictic time words and duration words suggests that these words are learned relationally (Tillman & Barner, 2015; Tillman et al., 2017; Wagner et al., 2016). In other words, rather than individually associating each word with perceptual representations of time or events, children initially learn time-word meanings by figuring out how they relate to other time words in the same semantic category. This account helps to explain why English-speaking children in prior studies demonstrated earlier understanding of the sequential order of deictic temporal terms (e.g., *next week* is after *tomorrow*) than knowledge of their remoteness (i.e., *next week* is approximately 7 days from now; Tillman et al., 2017). On this account, having access to different sets of time words could differentially impact German- and English-speakers’ acquisition even of the terms, like “yesterday,” that are lexicalized in both languages. For example, German’s inclusion of more tightly-spaced time words near “today” (e.g., vorgestern, gestern, heute, morgen, übermorgen) may help children triangulate meanings of proximal terms like *gestern*, sharpening category boundaries or providing semantic scaffolding. Conversely, German speakers might experience delays due to the larger overall set of terms they must master.

Moreover, differences in the English and German temporal lexicons might specifically impact children’s acquisition of particular facets of meaning. As discussed above, English-speakers appear to acquire adult-like meanings for temporal terms piecemeal, using different linguistic cues to learn different facets of their meaning (Tillman et al., 2017). If, for example, children use tense cues to learn deictic status, as prior studies in both English- and Hindi-speakers suggest (Maheshwari & Barner, 2025; Tillman et al., 2017), we would not expect to find developmental differences between English- and German-speakers in comprehension of the deictic status of terms like “yesterday,” given that both languages have past tense cues available. In contrast, acquisition of other facets of meaning, like order and remoteness, is more likely to be influenced by the presence of additional terms in the lexicon. For example, German-speaking children might be able to use the presence of “vorgestern” in the lexicon to help them pinpoint the remoteness of “gestern” to exactly one day from the present. The presence of additional single-word items (rather than phrases) might also facilitate German-speakers’ acquisition of the sequential order of time words, by providing more explicit lexical contrasts (i.e., the contrast between *morgen* and *übermorgen* may be clearer than that between *tomorrow* and *the day after tomorrow*). A summary table of relevant linguistic differences between English and German and their relation to our hypotheses about children’s comprehension of temporal terms is provided in the Supplementary Materials (Table S1).

To test these hypotheses, we recruited German- and English-speaking children aged 3–7 years to complete two spatial tasks: a timeline task (assessing knowledge of deictic status and sequential order; Tillman et al., 2017) and a novel calendar task (assessing knowledge of deictic status, temporal remoteness, and precise meanings). By comparing performance across languages, we aimed to evaluate the role of the temporal lexicon in shaping children’s understanding of individual facets of time meaning as well as their ability to integrate deictic status and remoteness to demonstrate knowledge of the precise definitions of “tomorrow” and “yesterday.”

## METHODS

### Overview

Children performed two primary tasks to test their time-word knowledge: the Timeline task and the Calendar task. In prior work, the Timeline task has been performed by children as young as age 3, and it has been used to independently assess children’s knowledge of deictic status, sequential order, and temporal remoteness (Tillman et al., 2017). In this task, as described further below, we presented children with a continuous horizontal line divided in half to indicate the past and future and with a midpoint representing the present (i.e., ‘now’). We evaluated participants’ 1) placement of temporal items (e.g., deictic time words) to the left vs. right of the midpoint and their 2) sequential ordering of these items along the timeline. Unlike prior studies, however, we did not use the timeline task to assess children’s knowledge of remoteness. Past work by Tillman et al. (2017) characterized children’s knowledge of temporal remoteness according to the strength of the relationship between children’s and adults’ timeline placements, after controlling for children’s understanding of order. Although it successfully measured remoteness knowledge separately from children’s knowledge of deictic status and order, this measure was nonetheless much more complex than the measures of the other facets of meaning, which were based on children’s proportions of correct responses. The reason for this difference is that there is no single spot on a continuous timeline—especially one whose endpoints are anchored to the lifespan of the participant using it—which consistently and objectively corresponds to the temporal remoteness of a given target. Given the overall complexity of this measure of remoteness, relative to the others, it is possible the timeline task may have underestimated children’s knowledge of this facet of time-word meaning. To address this possibility, we introduced a novel Calendar task to measure remoteness.

In the Calendar task, children placed stickers representing proximal (*yesterday* and *tomorrow*) and distal (*before-yesterday* and *after-tomorrow*^3^) terms in one of seven contiguous squares representing the days of the week. First, we scored whether children correctly placed items to the left or right of *today* (e.g., is *tomorrow* placed anywhere to the right of today, demonstrating knowledge of its future deictic status). Second, we scored whether children placed items the correct number of calendar squares from *today* (e.g., is *tomorrow* placed one square away from *today*, in either direction, demonstrating knowledge of its remoteness). Third, we scored whether children placed items in precisely the correct square (e.g., is *tomorrow* placed one square to the right of *today*, demonstrating knowledge of its precise meaning, combining both its deictic status and its remoteness). This task was not used to test children’s knowledge of sequential ordering, as each term was tested separately from the others. Together, these two tasks allowed us to separately assess children’s knowledge of all three facets of time-word meaning (deictic status, temporal remoteness, and order), as well as their knowledge of precise meanings.

The deictic terms and events assessed in the calendar and timeline tasks are presented in Table 1. In addition to assessing children’s knowledge of deictic status, order, temporal remoteness, and precise meanings of each term, we also examined the role of lexicalization and temporal distance by distinguishing between proximal (e.g., *yesterday*, *tomorrow*) and distal (e.g., *after-tomorrow*, *before-yesterday*) deictic terms. This distinction allowed us to evaluate whether children’s comprehension varied based on how temporally close or distant the referenced time point was from the present, and thus whether it was lexicalized in both languages or only in German.

**Table 1.** Temporal items placed on the Timeline and Calendar Tasks.

**Task**	**Item 1**	**Item 2**	**Item 3**	**Item 4**
Timeline - Event	breakfast	next birthday	dinner	last birthday
Timeline - Deictic 1	last week	tomorrow	tonight	this morning
Timeline - Deictic 2	next week	next year	yesterday	last year
Timeline - Deictic 3	yesterday	tomorrow	after-tomorrow	before-yesterday
Calendar	yesterday	tomorrow	before-yesterday	after-tomorrow

*Note*. In the Timeline task, participants placed four items on each line. In the Calendar task, each item was placed on a separate template. Timeline items are listed above in one of two orders participants were asked to mark their locations; half the participants received the items in the reverse order from that shown above. Calendar items are listed in one of four counterbalanced orders participants were asked to place stickers on templates. Items “the day before yesterday” and “the day after tomorrow” are abbreviated to *before-yesterday* and *after-tomorrow*, respectively. Results from Deictic Timeline 3 and the Calendar task are discussed in the main text, while results from all timelines are reported in the Supplementary Materials (see Supplementary Figures S2, S3, and Table S12).

### Participants

The final analyses included data from 151 English-speaking children (*M*_age_ = 5;3; range = 3;2–7;11) and 153 German-speaking children (*M*_age_ = 5;1; range = 3;1–7;11). This sample-size was chosen to exceed that of prior developmental studies using similar tasks (e.g., Tillman et al., 2017 tested *n* = 117 children between 3 and 8 years), while remaining feasible given time limitations at testing sites. English-speaking children were recruited in the United States in Austin, Texas and included *n* = 22 3-year-olds (*M*_*age*_ = 44 months, range 38–47 months), *n* = 47 4-year-olds (*M*_*age*_ = 53 [48–59]), *n* = 42 5-year-olds (*M*_*age*_ = 65 [60–71]), *n* = 28 6-year-olds (*M*_*age*_ = 78 [73–83]), and *n* = 12 7-year-olds (*M*_*age*_ = 91 [85–95]). German-speaking children were recruited in Vienna, Austria, and included *n* = 38 3-year-olds (*M*_*age*_ = 43 months, range 37–47 months), *n* = 42 4-year-olds (*M*_*age*_ = 54 [48–59]), *n* = 39 5-year-olds (*M*_*age*_ = 66 [60–71]), *n* = 18 6-year-olds (*M*_*age*_ = 76 [72–83]), and *n* = 16 7-year-olds (*M*_*age*_ = 90 [84–95]). To establish a developmental benchmark, we also tested adult controls: *n* = 45 English-speaking adults and *n* = 30 German-speaking adults. Analyses involving adult controls are reported in the Supplementary Materials. Participants were tested in their native language by experimenters who were also native speakers of that language and both samples were drawn from childcare centers in urban areas that primarily serve middle-class families. Several child participants in our final sample were bilingual (*n* = 24 English speakers; *n* = 22 German speakers), but multilingualism did not impact any of our results (see Supplementary Materials for analyses and more details about bi-/multilingual participants).

We excluded an additional 34 English-speaking participants from analyses due to having missing data on the timeline task (*n* = 12 3-year-olds, *n* = 3 4-year-olds, *n* = 5 5-year-olds, and *n* = 2 6-year-olds), having missing data on the calendar task (*n* = 1 4-year-old), having primary language spoken at home that was not English (*n* = 3 5-year-olds, *n* = 1 6-year-old), or experimenter error (*n* = 3 4-year-olds, *n* = 3 5-year-olds, *n* = 1 6-year-olds, and *n* = 1 adult). We also excluded six German-speaking 3-year-old participants due to having missing data on the Timeline task.

### Materials and Procedure

We tested participants one-on-one using an identical procedure for both children and adult controls. The experimenter began the session by asking the participant to recite the days of the week. Next, participants completed the Timeline and Calendar tasks. Task order was counterbalanced across participants. Finally, participants answered five verbal forced-choice questions about the calendar system: “Which day comes after today: tomorrow or yesterday?”, “Which day comes before today: tomorrow or yesterday?”, “What day of the week is it today?” and “Today is [correct day of the week]. What day of the week was it yesterday?” and “Today is [correct day of the week]. What day of the week will it be tomorrow?” All verbal questions were coded as correct (1) or incorrect (0). Due to missing data (*n* = 20 German-speaking 3-year-olds), the results of the verbal questions for 3-year-olds are excluded from primary analyses, and we only present the results for 4- through 7-year-olds.

### Timeline Task Procedure

**Figure 1.**
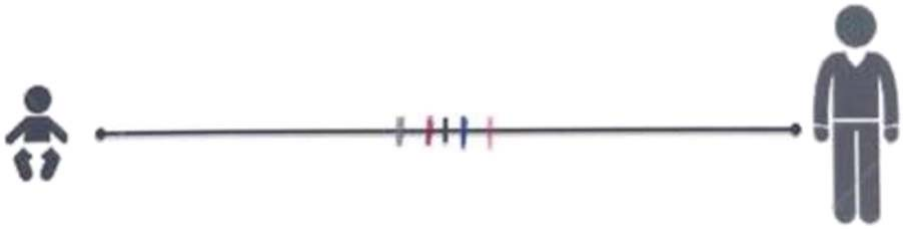
Timeline template showing a 6-year-old child’s placement of ‘tomorrow’ (blue line), ‘yesterday’ (red line), ‘before-yesterday’ (grey line), and ‘after-tomorrow’ (pink line). *Note*. On each timeline, there was a black line in the middle bisecting the line to represent ‘now, ’ and participants indicated the placement for each target item (e.g., *tomorrow*, here in blue) with a pencil mark.

Adapted from Tillman et al. (2017), the Timeline task assessed participants’ understanding of the deictic status and order of deictic terms and temporal events (see Table 1). Study materials included seven colored pencils (blue, red, pink, brown, orange, grey, and green) and a sheet of paper containing four continuous horizontal timelines arranged in a vertical column. The center of each timeline was bisected with a small black vertical mark; left and right endpoints were marked by small black dots. A silhouette of a baby was positioned to the left of the timeline and an adult silhouette to the right, representing time in the past and future, respectively (see Figure 1).

The experimenter placed a sheet with four blank timelines in front of the participant and described the first timeline as showing “when different things happen,” going from the past, “when you were a baby,” to the future, “when you’re going to be a grown-up,” with the present, “right now”, represented by a small vertical mark in the middle. The experimenter asked the participant to indicate, with a pencil mark, where each item goes on the line (e.g., “When did you eat breakfast today? Draw a line for when you ate breakfast today”). If children (e.g., 3-year-olds) had difficulty following the instructions to make marks on the line, they were instead asked to point to the line to indicate where each target item belongs on the line and the experimenter made the pencil mark with the appropriate colored pencil.

Each timeline was associated with a predetermined list of four items (Table 1) and participants indicated the location of four items on each of the three timelines. The experimenter began each set of four trials with a blank timeline, covering the preceding timeline with a blank sheet of paper.^4^ Participants always received the ‘Event’ line first, followed by the three ‘Deictic’ lines. For each line, half of the participants received the items in the order shown in Table 1 (Order 1; from item 1 to item 4), and the rest received them in the reverse order (Order 2; from item 4 to item 1). Our use of two Orders and the specific ordering of items tested within each was determined so as to provide a variety of different trial-by-trial contrasts in deictic status, sequential order, and remoteness.

To focus on the critical linguistic difference between English and German, in the main text we report only analyses of performance on Deictic Timeline 3, containing the terms before-yesterday/yesterday/tomorrow/after-tomorrow. Analyses of performance on the ‘Event’ timeline (which served as a control), and performance across the larger set of deictic terms in all three Deictic timelines, are reported and discussed in the Supplementary Materials.

### Timeline Coding

After testing was complete, a researcher measured the distance (rounded to the nearest half mm) from each of the participant’s marks representing temporal items to the line bisecting the midpoint of the timeline (0). Distances of items placed to the left of the midpoint were coded with negative values, and items placed to the right were coded with positive values. To assess knowledge of deictic status on the Timeline task, we coded whether the response to each item was located to the right or left of the midpoint (irrespective of its exact distance in mm). For example, a response for *yesterday* was coded as correct (1) if it was marked to the left of the midpoint of the line. To assess knowledge of sequential order, separate from deictic status, we coded whether each response (i.e., on trial N) was correctly located to the left or right of their previous response (trial N − 1) on the same timeline. This “1-back” order coding approach was previously used by Tillman et al. (2017), who found that it yielded similar patterns of results to coding the rank-ordering of all four items on the timeline, while avoiding penalizing younger children for potential limitations in working memory. In order to succeed on a given trial, children only needed to recall the meaning of their most recent mark on the line. For example, for a child who received Order 1, a response for *tomorrow* (item 2 on Deictic timeline 3, see Table 1) was coded as correct (1) if it was placed to the right of their response for *yesterday* (the previous item), disregarding the item’s location relative to the present (indicated by the midpoint).

### Calendar Task Procedure

**Figure 2.**
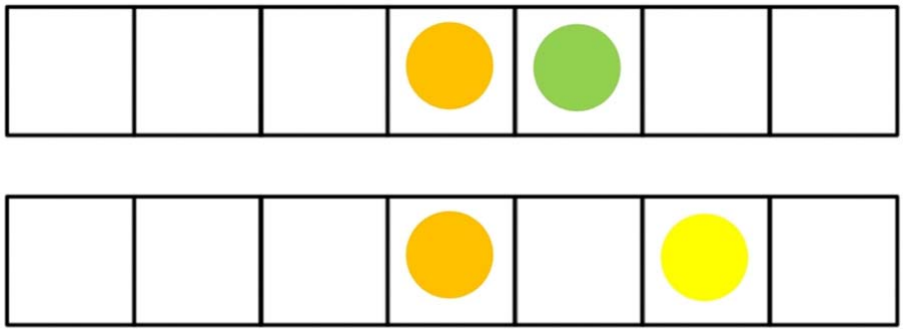
Calendar Templates and Correct Sticker Placement for ‘tomorrow’ (top) and ‘after-tomorrow’ (bottom). *Note*. On each trial, the orange sticker represented *today*, and participants selected the square placement for the target item, e.g., *tomorrow* (here shown in green).

The Calendar task is a novel measure in which participants’ responses are constrained to seven discrete squares with precise distances from ‘today. ’ Like the Timeline task, the Calendar task also tests participants’ knowledge of each target word’s deictic status (e.g., that *yesterday* is in the past). Unlike the Timeline task, the Calendar task also provides a precise test of participants’ knowledge of the remoteness of each term (e.g., that *yesterday* is exactly one day from *today*). Study materials included a sheet of paper containing eight rows of seven contiguous squares (see Figure 2 for an example) and colored round label stickers (blue, red, green, yellow, and orange). Each row of squares represents time extending from three days in the past to three days in the future, relative to *today* (square 4). The correct squares for *before-yesterday*, *yesterday*, *tomorrow*, and *after-tomorrow* were 2, 3, 5, and 6, respectively.

Participants were first asked to name the days of the week (“Do you know the days of the week? Can you say them for me?”). The experimenter recorded whether the participant was able to do so without assistance, and, if not, prompted them by beginning to recite the list with rising intonation, e.g., “Monday, Tuesday …” Next, to begin the Calendar task, the experimenter placed a sheet of eight rows of seven contiguous squares in front of the participant. In order to familiarize the participant with the conventional left-to-right calendar layout in which each day is represented by a box, the experimenter said, “Look, these boxes are for the days of the week,” and then pointed to each square in the topmost template from left to right, and labelled each one with a day of the week. Next, the experimenter said, “Okay, now can you tell me what day this box is for?” and asked the participant to verbally label each square with a day of the week, while the experimenter also named the days in unison with the participant. This verbal labeling of the calendar template began with the day—either Sunday or Monday—that the participant had previously started with when asked to recite the days of the week in the absence of a template (prior to starting the paper-based tasks), and the experimenter provided feedback if the child’s labelling was incorrect.

After this familiarization phase, the experimenter began each test trial with an empty row of squares, covering the row of squares from the preceding trial with a blank sheet of paper. On each trial, the experimenter placed the orange sticker in the center (in square 4) to represent *today* (“Let’s pretend that this sticker is for today”), then gave the participant a sticker to place in another square (e.g., “If this one is *today*, can you put the green sticker in the square for *tomorrow*?”). Different colored stickers were used for each target: yesterday (blue), tomorrow (green), after-tomorrow (yellow), and before-yesterday (red). If the participant’s initial sticker placement was correct, that row of squares was covered with a sheet of paper so that they could no longer see it, and the participant was prompted to place the next target item on the next blank row of squares. If the participant did not place a sticker in the correct square on the first try, their initial response was covered, and on the next blank row of squares they were given a forced-choice between the proximal and distal squares on the correct side of *today*, e.g., in the case of *tomorrow*: Is it this one [experimenter points to square 6] or this one [experimenter points to square 5]. Although children received no overt feedback on whether either their initial or second sticker placements were correct, the inclusion of these follow-up forced choices may have provided an additional cue to children that they should consider temporal distance (i.e., remoteness) when placing their stickers. Participants’ follow-up choices were recorded but not included in the primary analyses. All data reported in the Results reflect the participants’ initial sticker placements on each trial. The trial order was counterbalanced across subjects, who each received one of four different Orders, and each target item was tested on a separate row without visual access to previous sticker placements.

### Calendar Task Coding

To assess knowledge of precise meanings on the Calendar task, we coded whether participants placed the sticker in the correct square. For example, a sticker placement for ‘tomorrow’ was coded as correct (1) if it was placed in square 5, and as incorrect (0) if it was placed in any other square.

To assess knowledge of deictic status on the Calendar task, we coded whether participants correctly placed stickers to the right or left of the correct box for *today*. For example, if the sticker for *tomorrow* was placed in any square 5–7, it was coded as correct (1), and if it was placed in any square 1–3, it was coded as incorrect (0).

To assess knowledge of remoteness on the Calendar task, we coded whether participants placed stickers the correct distance from *today* in either direction on the first try. For example, a sticker placement for *tomorrow* was coded as correct (1) if it was placed in square 3 or 5 and incorrect (0) if it was placed in squares 1–2 or 6–7.

### Analysis

We ran mixed-effects logistic regressions in the *lme4* package in *R* (Bates et al., 2015). Age was a continuous, scaled variable in all models. We included random intercepts for subjects in all models, and all fixed effects were coded with predictors centered around 0 [−1, 1]. We performed Wald chi-square tests from type-III analysis-of-variance tables using the *car* package (Fox & Weisberg, 2019) to determine whether models including each factor of interest provided a significantly better fit to the data than reduced models. Adult data were omitted from all predictive models and are discussed in the Supplementary Materials. Post hoc analyses comprised one-sided Wilcoxon signed-rank tests to compare performance to chance levels and two-sample Wilcoxon rank-sum tests to compare English- and German-speaking children’s performance at each age. Full results for all post-hoc analyses, including non-significant comparisons, are reported in the Supplementary Materials.

For all post-hoc analyses, we also conducted post-hoc power analyses using the pwr package in R (Champely, 2020). For each one-sample contrast against chance, achieved power was based on the estimated effect size (*r*), sample size (*n*), and a two-tailed test with *α* = .05. These analyses are intended to contextualize both significant and non-significant results, and we acknowledge the limitations of post-hoc power estimation.

To provide a clearer sense of which effects could be reliably detected for the two-sample Wilcoxon rank-sum tests comparing English- and German-speaking children, we conducted sensitivity power analyses using the *pwr* package in R. These analyses estimate the Smallest Effect Size of Interest (SESOI) that could be reliably detected with 80% power (*α* = .05). Results indicate that, for smaller subgroups, small-to-moderate effects could be reliably detected. These SESOI values are reported in Supplementary Materials (Tables S3 and S4).

Analysis code, as well as de-identified data and task materials (e.g., experimenter scripts) are available on the Open Science Framework: https://osf.io/4m9zt/.

## RESULTS

Table 2 provides a summary table of our primary findings, which are discussed in detail in Knowledge of Precise Meanings, Knowledge of Deictic Status, Knowledge of Sequential Order, and Knowledge of Remoteness sections. Additional analyses appear in the Supplementary Materials.

**Table 2.** Summary of cross-linguistic differences in task performance.

**Measure**	**Task**	**Language Effect**	**Age Effect**	**Item Type Effect**	**Interactions**
Precise Meaning	Calendar	Yes; German > English	Yes	Yes; Proximal > Distal	None
Deictic Status	Calendar	No	Yes	No	Item Type × Age: Proximal > Distal at Age 6
	Timeline	No	Yes	No	None
Order	Timeline	No	Yes	No	Language Group × Age: German > English at Age 3
Remoteness	Calendar	Yes; German > English	Yes	Yes; Proximal > Distal	None

*Note*. The primary analysis for each measure was a mixed-effects model that included fixed effects of Language Group, Age, Item Type, and all possible interactions.

### Knowledge of Precise Meanings

#### Calendar Task.

To assess children’s knowledge of the precise meaning of each temporal term—including knowledge of both its deictic status and its remoteness—we coded whether participants placed the corresponding sticker in the correct square on the Calendar task (on the first try). We then asked whether correct sticker placements were predicted by children’s Language Group (English vs. German), their Age, the Item Type (Proximal terms lexicalized in both languages vs. Distal terms lexicalized only in German). In this model, we also included all possible two- and three-way interactions between these factors. The model revealed significant main effects of Language Group (*β* = −0.297, *p* = .01; *χ*^2^(1) = 6.59, *p* = .01), Age (*β* = 1.509, *p* < .001; *χ*^2^(1) = 119.70, *p* < .001) and Item Type (*β* = −0.763, *p* < .001; *χ*^2^(1) = 80.44, *p* < .001). However, none of these main effects were qualified by an interaction. Overall, older children outperformed younger children, children were more likely to have precise meanings for proximal terms than distal ones, and German-speakers out-performed English-speakers. Critically for the present study, the main effect of Language Group combined with the lack of a two-way interaction between this factor and Item Type suggests that German speakers had an advantage for *both* the distal terms that are lexicalized only in German and the proximal ones that are lexicalized in both languages.

**Figure 3.**
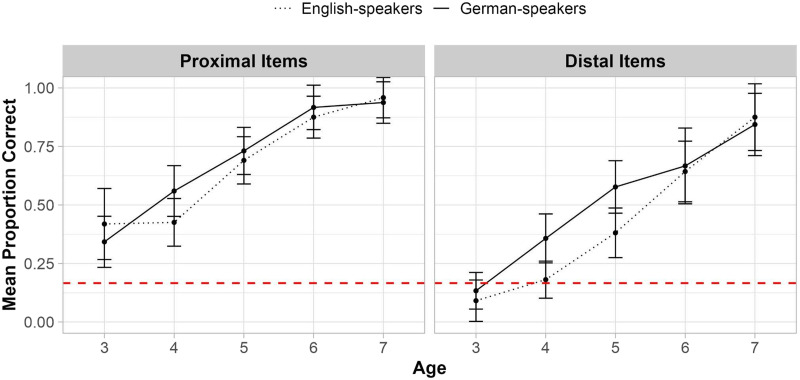
Precise meaning accuracy (%) on the Calendar Task. *Note*. ‘Proportion Correct’ indicates the mean proportion of items German-speaking children (*n* = 153) and English-speaking children (*n* = 151), grouped by age (in years), placed in the correct square, averaged across proximal (left) and distal (right) terms. Error bars represent 95% confidence intervals and the red dashed line marks chance performance per trial (.167, or 1 of 6 possible squares).

Figure 3 shows the mean precise meaning accuracy for proximal and distal terms as a function of age for each language group. See Supplementary Table S2 for mean accuracy for individual terms (e.g., “yesterday”) and age groups. Interestingly, visual examination of the data in Figure 3 suggests that the German advantage for the proximal terms lexicalized in both languages peaks around the age of 4, while the advantage for distal terms lexicalized only in German persists longer, until age 6. However, as discussed above, we did not observe a statistically significant interaction between Language Group and Age, and we do not have sufficient statistical power to detect effects of language within all individual age groups in the current study (see Supplementary Table S3). We therefore discuss implications for future research examining the developmental time-course of the German advantage in the General Discussion.

To assess whether children’s overall performance exceeded chance, we conducted one-sided Wilcoxon signed-rank tests. Chance on this measure was 1/6, because children could put their stickers in one of 6 possible squares, only one of which was correct. Performance for proximal terms was significantly above chance at age 3 for English-speakers (*V* = 660, *p* < .001, *r* = 0.856) but not until age 4 for German-speakers (for 3-year-olds: *V* = 152, *p* = .159, *r* = 0.0375; for 4-year-olds: *V* = 2,867, *p* < .001, *r* = 0.527). For distal terms, above-chance performance emerged at age 5 for both German-speakers (*V* = 2,520, *p* < .001, *r* = 0.552) and English-speakers (*V* = 2,192, *p* = .030, *r* = 0.198).^5^ See Supplementary Table S4 for complete results from all one-sided Wilcoxon signed-rank tests.

Having found that German-speaking children have increased comprehension of the precise meanings of deictic temporal terms relative to English-speakers, we next asked about the sources of those differences. In other words, what specific aspects of time-word meaning do German-speakers acquire earlier or more easily? To address this question, the next analyses asked whether German- and English-speaking children differed in their knowledge of three individual components of temporal meaning: deictic status, sequential order, and remoteness.

### Knowledge of Deictic Status

#### Timeline Task.

To assess a participant’s overall deictic status accuracy (i.e., knowledge of whether a term pertains to the past or the future), we calculated the percentage of trials on which each participant placed past items to the left of the midpoint of the timeline and future items to the right.

**Figure 4.**
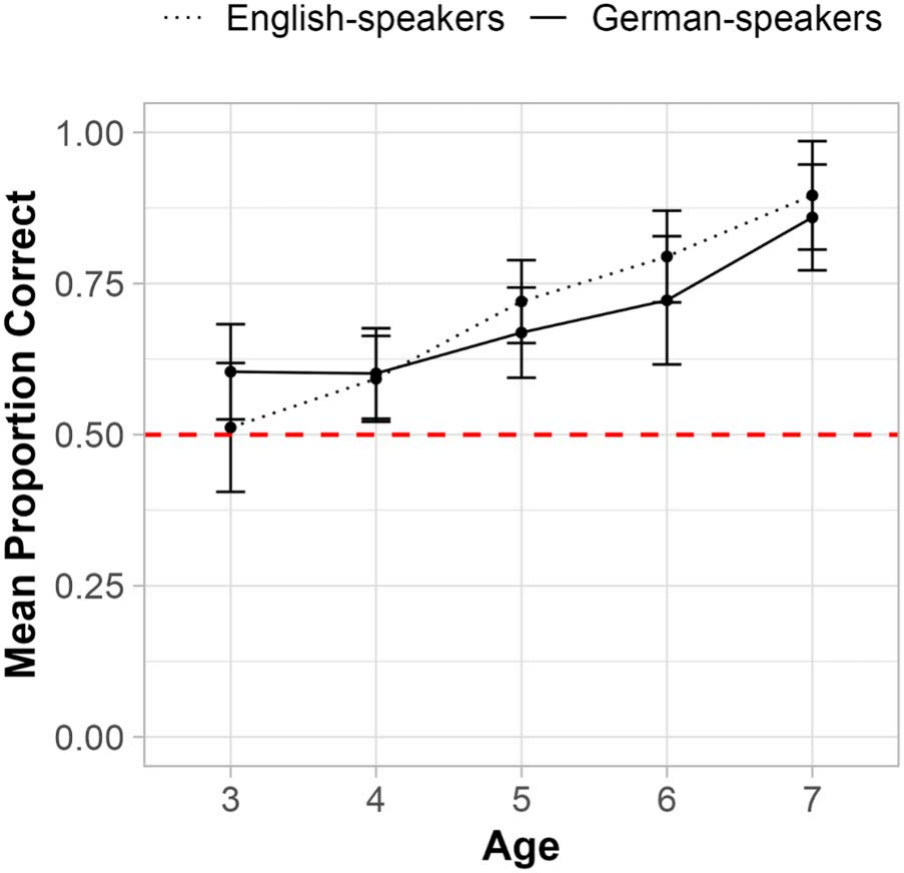
Deictic Status Knowledge on the Deictic Timeline 3. *Note*. ‘Proportion Correct’ indicates the proportion of correct placements in the past or future for items on Deictic Timeline 3 (see Table 1) by participants in each Language Group (*n* = 153 German speakers, *n* = 151 English speakers) and by age (in years). Error bars represent 95% confidence intervals. The dashed red line indicates chance performance per trial (at 50%).

We constructed a model to predict correct placement of terms in the past vs. future from Language Group (German vs. English), Age, Item Type (Proximal vs. Distal), and all possible two- and three-way interactions. We restricted our primary analysis to the Deictic 3 Timeline (see Table 1) so that we could compare placements of proximal terms (*yesterday*, *tomorrow*) that are lexicalized in both languages to the placements of distal terms (*after-tomorrow*, *before-yesterday*) that are lexicalized only in German. The model revealed a significant effect of Age (*β* = 0.477, *p* < .001; *χ*^2^(1) = 40.50, *p* < .001; see Figure 4) but no significant effect of Language Group (*β* = 0.020, *p* = .765; *χ*^2^(1) = 0.004, *p* = .947) or Item Type (*β* = −0.13, *p* = .844; *χ*^2^(1) = 0.047, *p* = .829), suggesting that German speakers did not have increased knowledge if the deictic status of *either* the distal terms that are lexicalized only in German or the proximal ones that are lexicalized in both languages. Results from models including data from all three ‘Deictic’ timelines, as well as the data from the control ‘Event’ line are reported in the Supplementary Materials. In all cases, only the effect of Age was significant.

To assess whether children’s overall performance exceeded chance on Deictic Timeline 3, we conducted one-sided Wilcoxon signed-rank tests comparing performance to chance (see Supplementary Table S8). Chance on a single trial is 50% because correct responses included any location to the left of the midpoint for past items and any location to the right of the midpoint for future items. Performance on Deictic Timeline 3 was above chance by age 3 for German-speakers (*V* = 6,750, *p* < .001, *r* = 0.140) but not until age 4 for English-speakers (for 3-year-olds, *V* = 1,827.5, *p* = .415, *r* = −0.058; for 4-year-olds: *V* = 10,082.5, *p* = .006, *r* = 0.117).

#### Calendar Task.

**Figure 5.**
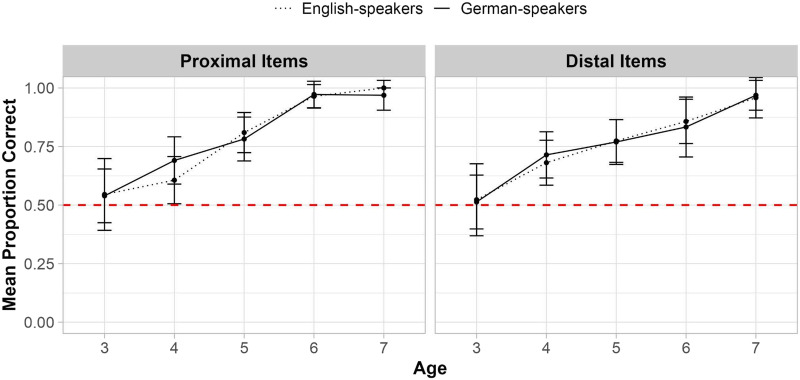
Deictic Status Knowledge on the Calendar Task. *Note*. ‘Mean Proportion Correct’ indicates the average proportion correct (*n* = 153 German-speakers, *n* = 151 English-speakers) of placements in the past or the future on the Calendar task by age (in years) and Item Type (Proximal vs. Distal). We calculated the proportion correct from individuals’ average performance across two proximal terms (*yesterday*, *tomorrow*) and two distal terms (*after-tomorrow*, *before-yesterday*) separately. The red dashed line represents chance performance per trial (50%).

Next, we asked how children’s knowledge of deictic status was reflected in their performance on the Calendar task. We constructed a model to test if Language Group (German vs. English), Age, Item Type (Proximal vs. Distal) and all possible two- and three-way interactions predicted correct placement of stickers in the past vs. future squares. The model revealed a significant effect of Age (*β* = 1.02, *p* < .001; *χ*^2^(1) = 88.47, *p* < .001; see Figure 5) and a two-way interaction between Item Type and Age (*β* = −0.175, *p* = .055; *χ*^2^(1) = 3.69, *p* = .055). We did not detect a significant effect of Language Group (*β* = −0.024, *p* = 0.790) or any other interactions. To further investigate the interaction between Item Type and Age, we conducted follow-up two-sample Wilcoxon rank-sum tests comparing children’s performance on proximal and distal terms at each age group. At age 6, children’s performance was significantly higher for proximal trials (*M* = 97% accuracy, *SD* = 18% accuracy) relative to distal trials (*M* = 85% accuracy, *SD* = 36% accuracy; *W* = 3,726, *p* = .005, *r* = −0.103), but no other statistically significant differences arose, possibly due to limited power (see Supplementary Table S6).

To assess whether children’s performance exceeded chance, we conducted one-sample Wilcoxon signed-rank tests comparing performance to a chance level of 50% for proximal and distal terms separately. Performance was above chance by age 4 in both language groups for both proximal terms (German: *V* = 2,465, *p* < .001, *r* = 0.331; English: *V* = 2,708, *p* = .020, *r* = .185) and distal terms (German: *V* = 2,550, *p* < .001, *r* = 0.372; English: *V* = 3,040, *p* < .001, *r* = .314).^6^ For comprehensive results see Supplementary Table S5.

#### Timeline and Calendar Task Comparison.

Most critically for the questions of the present study, the results presented so far do not show differences in deictic status knowledge between German and English speakers on either the Calendar task or the Timeline task. Because we were secondarily interested in task differences, we also compared children’s knowledge of the deictic status of the same items when tested on each task (Calendar vs Deictic Timeline 3, see Table 1). Given the absence of a main effect of Language Group in previous analyses of deictic status accuracy, we examined whether Age, Task (Calendar vs. Timeline), or their two- and three-way interactions predicted children’s accuracy in placing items in the past or future. The model revealed a significant main effect of Age (*β* = 0.482, *p* < .001; *χ*^2^(1) = 34.77, *p* < .001) and a two-way interaction between Task and Age (*β* = 0.696, *p* < .001 , *χ*^2^(1) = 90.61, *p* < .001).

Follow-up one-sided Wilcoxon signed-rank tests comparing performance to a chance level of 50% revealed above-chance performance at age 3 on the Timeline task (*V* = 15,561.0, *p* = .015, *r* = 0.066), but not until age 4 on the Calendar task (for 3-year-olds: *V* = 15,303.5, *p* = .183, *r* = 0.051; for 4-year-olds: *V* = 42,661.5, *p* < .001, *r* = 0.297). Follow-up two-sample Wilcoxon rank-sum tests comparing performance on the Calendar and Timeline tasks at each age did not find significant differences in 3-year-olds’ performance on the Calendar task (53% accuracy) and Timeline task (57% accuracy; *W* = 26,795.5, *p* = .363, *r* = −0.060).^7^ By age 4, children’s performance was significantly higher on the Calendar task (67% accuracy) relative to the Timeline task (59% accuracy, *W* = 67,340.0, *p* = .039, *r* = 0.054). This pattern continued at age 5 (*M*_calendar_ = 78%, *M*_timeline_ = 69%, *W* = 56770.0, *p* = .011, *r* = 0.071), age 6 (*M*_calendar_ = 91%, *M*_timeline_ = 77%, *W* = 19,320.0, *p* < .001, *r* = 0.122), and age 7 (*M*_calendar_ = 97%, *M*_timeline_ = 88%, W = 6,888.0, *p* = .0067, *r* = 0.085).^8^

### Knowledge of Sequential Order

#### Timeline Task.

**Figure 6.**
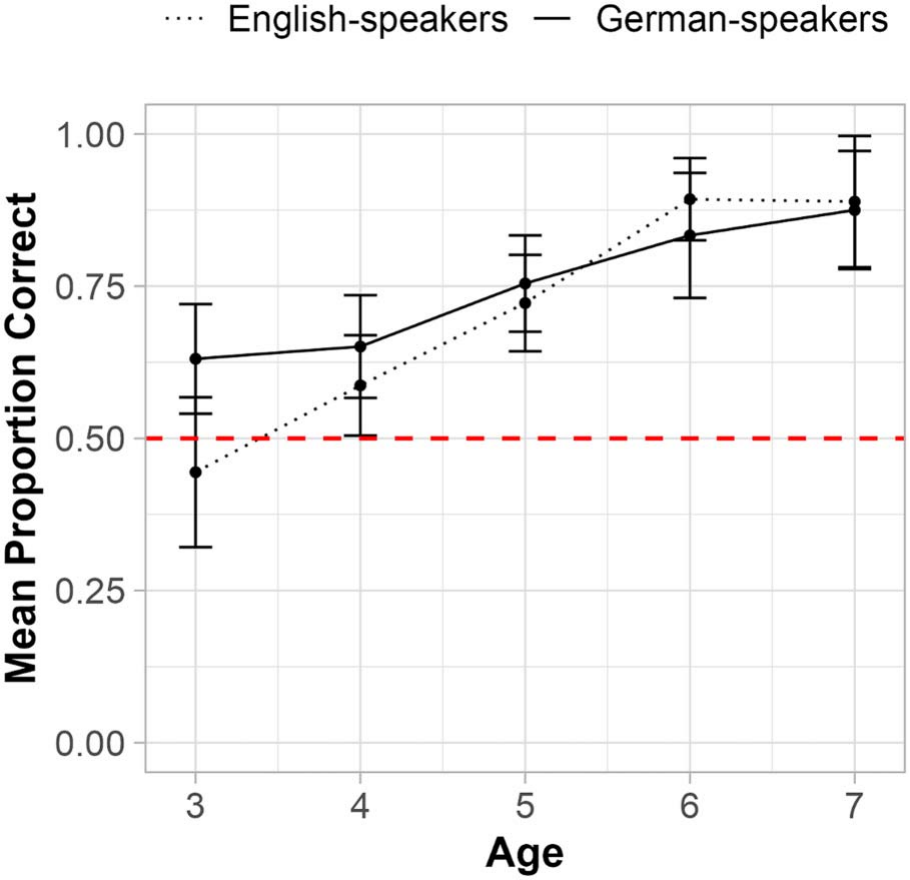
Order Knowledge on the Deictic 3 Timeline 3. *Note*. ‘Proportion Correct’ indicates the proportion of correct placements in sequential order for items on Deictic Timeline 3 (see Table 1) by participants in each language group (*n* = 153 German speakers, *n* = 151 English speakers) and by age (in years). Error bars represent 95% confidence intervals. The dashed red line indicates chance performance per trial (at 50%).

We next assessed children’s knowledge of the sequential ordering of deictic terms on the Timeline task by comparing children’s placement of each term on the timeline to their placement of the immediately preceding term. We restricted our primary analyses to the Deictic Timeline 3 (see Table 1) in order to compare placements for proximal term time words (*yesterday*, *tomorrow*) lexicalized in both languages to the placements of distal terms (*after-tomorrow*, *before-yesterday*) lexicalized only in German^9^. Results from all three ‘Deictic’ timelines as well as results from the control ‘Event’ timeline are reported in the Supplementary Materials. Figure 6 shows mean sequential order knowledge for proximal vs. distal terms as a function of age for each language group.

We constructed a model to predict knowledge of sequential order from Language Group (German vs. English), Age, Item Type (Proximal vs. Distal) and all possible two- and three-way interactions. The regression coefficient for Age was significant (*β* = 0.833, *p* < .001; *χ*^2^(1) = 44.31, *p* < .001). The model also revealed a significant two-way interaction between Language Group and Age (*β* = −0.383, *p* = .0342; *χ*^2^(1) = 4.48, *p* = .033). There was no significant effect of Item Type (*β* = −0.157, *p* = 0.164; *χ*^2^(1) = 0.34, *p* = 0.560) or significant two- or three-way interactions.

To further investigate the interaction between Language Group and Age, we conducted follow-up two-sample Wilcoxon rank-sum tests comparing performance between language groups at each age tested. These tests revealed that German-speakers out-performed English-speakers only at age 3 (*W* = 2,845.5, *p* = .018, *r* = −0.203; but see Supplementary Table S10 for comprehensive results from all two-sample Wilcoxon rank-sum tests).^10^ Moreover, follow-up one-sided Wilcoxon signed-rank tests comparing performance to a chance level of 50% revealed that at age 3, German-speakers performed above chance (*V* = 3,920.0, *p* = .003, *r* = 0.170), while English-speaking children did not do so until age 4 (for 3-year-olds: *V* = 896, *p* = .812, *r* = −0.165; for 4-year-olds, *V* = 5,629.5, *p* = .021, *r* = 0.108).^11^ See Supplementary Table S9 for comprehensive results from all one-sided Wilcoxon signed-rank tests.

### Knowledge of Remoteness

#### Calendar Task.

**Figure 7.**
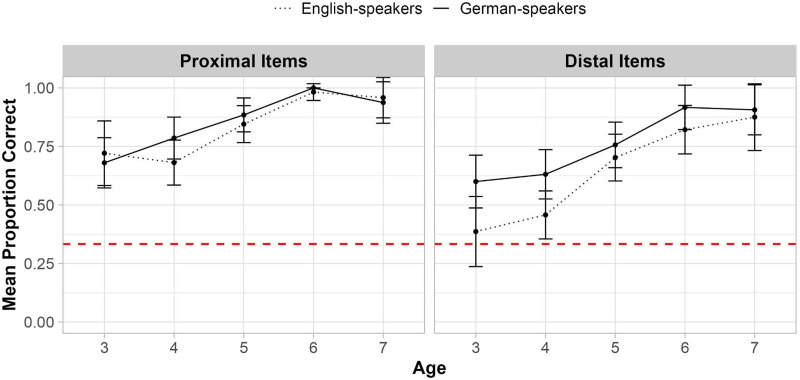
Knowledge of Temporal Remoteness on the Calendar Task. *Note*. ‘Mean Proportion Correct’ indicates the average proportion correct (*n* = 153 German- speakers, *n* = 151 English- speakers) by age (in years) who placed proximal (left) and distal (right) terms the correct distance from *today*. Error bars represent 95% confidence intervals, and the dashed line is chance performance (1/3).

We next assessed children’s understanding of temporal remoteness, this is, how distant each term is from today, irrespective of its deictic status and ordering relative to other terms. We constructed a model predicting children’s knowledge of the remoteness of terms on the Calendar task from Language Group (German vs. English), Age, Item Type (Proximal vs. Distal), and all possible two-and three-way interactions. The model revealed main effects of Language Group (*β* = −0.231, *p* = .006; *χ*^2^(1) = 13.60, *p* < .001), Age (*β* = 0.917, *p* < .001; *χ*^2^(1) = 132.17, *p* < .001), and Item Type (*β* = −0.488, *p* < .001; *χ*^2^(1) = 40.82, *p* < .001), but no significant two- or three-way interactions. As shown in Figure 7, German-speakers outperformed English-speakers; performance improved with age; and children had more knowledge overall about proximal terms than distal terms^12^.

We also conducted one-sided Wilcoxon signed-rank tests comparing performance to a chance level of 33.33% at each age (see Supplementary Table S7). Chance here is 1/3 because there were six possible sticker placements and two possible correct placements (i.e., correct distance from today in either direction). Performance was above chance for proximal terms by age 3 in both language groups (German: *V* = 2,550, *p*, .001, *r* = 0.646; English: *V* = 868, *p* < .001, *r* = 0.656). For distal terms, above chance performance emerged at age 3 among German-speaking children (*V* = 2,385, *p* < .001, *r* = 0.548) but not until age 4 among English-speaking children (for 3-year-olds: *V* = 612, *p* = .079, *r* = 0.206; for 4-year-olds: *V* = 3,139, *p* < .001, *r* = 0.353).

### Verbal Knowledge Performance

Our primary tasks testing children’s knowledge of deictic terms—the Timeline and Calendar tasks—were both spatial in nature. We were also interested in whether we would see language-group differences in general knowledge of the calendar system on a non-spatial task. We therefore also asked children a series of verbal questions, all of which were related to time.

#### Knowledge of the Days of the Week.

When asked to recite the days of the week, 30% of German-speakers (including 0% of 3-year-olds, 6% of 4-year-olds, 35% of 5-year-olds, 28% of 6-year-olds, and 88% of 7-year-olds) and 36% of English-speakers (including 13% of 3-year-olds, 13% of 4-year-olds, 48% of 5-year-olds, 48% of 6-year-olds, and 92% of 7-year-olds) did so on their first try without assistance or feedback from the experimenter. Age (in years), *β* = 1.49, *p* < .001; *χ*^2^(1) = 53.54, *p* < .001, Language Group, *β* = 0.947, *p* < .001; *χ*^2^(1) = 30.43, *p* < .001, but not their interaction (*β* = 0.022, *p* = .917; *χ*^2^(1) = 0.011, *p* = .917), predicted children’s ability to recite the days of the week without prompting. Performance by speakers of both languages improved with age and follow-up two-sample Wilcoxon rank-sum tests revealed that and English-speakers were more likely than German-speakers to correctly recite the days of the week in order at age 4 (*W* = 1,123, *p* < .001, *r* = 0.377), 5 (*W* = 1,077, *p* < .001, *r* = 0.428), and 6 (*W* = 371, *p* = .002, *r* = 0.460), but not at age 3 (*W* = 69, *p* = .350, *r* = 0.194) or age 7 (*W* = 108, *p* = .232, *r* = 0.236).

#### Verbal Knowledge of Yesterday and Tomorrow.

**Figure 8.**
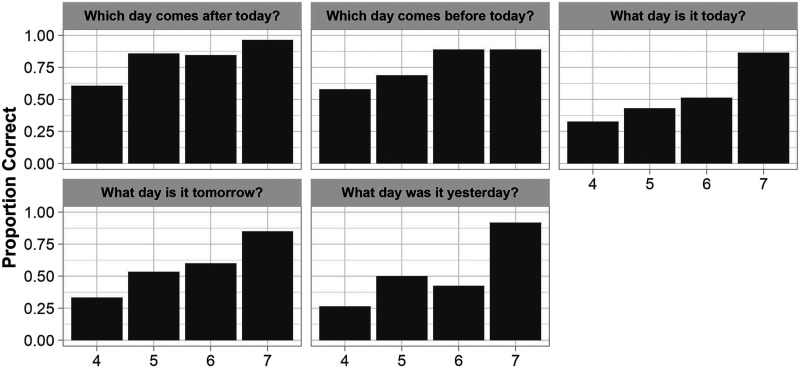
Proportion Correct on Verbal Questions at Each Age Tested. *Note*. ‘Proportion Correct’ represents the percentage of children (collapsed across language groups) who correctly answered each verbal question.

We also asked children two verbal forced-choice questions about the meanings of *yesterday* and *tomorrow*, and three open-ended questions about how these terms apply to the days of the week (see Figure 8). Three-year-olds were excluded from these analyses due to a high rate of missing responses in this age group (i.e., 91% of German-speaking three-year-olds and 42% of English-speaking 3-year-olds). We tested whether children’s performance was predicted by Age, Item, Language Group, and all two- and three-way interactions between these factors. We found that performance on the two forced-choice questions (e.g., “Which day comes before today: *yesterday* or *tomorrow*?”) improved with Age (*β* = 1.26, *p* = .005; *χ*^2^(1) = 14.42, *p* < .001), but neither Item (*β* = −0.368, *p* = .372; *χ*^2^(1) = 3.655, *p* = .056), Language Group (*β* = 1.11, *p* = .092; *χ*^2^(1) = 0.918, *p* = .338), or possible two- and three-way interactions predicted performance. As shown in Figure 8, performance on the verbal forced-choice questions improved monotonically with age (*M*_4_ = 0.59 < *M*_5_ = 0.75 < *M*_6_ = 0.90 < *M*_7_ = 0.93), with above-chance performance emerging by age 4 (*V* = 8,316, *p* = .008, *r* = 0.161).

Performance on the three open-ended questions assessing children’s verbal knowledge of the days of the week (e.g., “Today is [Tuesday]. What day was it yesterday?”) also improved with Age (*β* = 1.29, *p* < .001; *χ*^2^(1) = 28.22, *p* < .001; see Figure 8). Language Group (*β* = 0.378, *p* = .252; *χ*^2^(1) = 0.835, *p* = .361) Item (*χ*^2^(1) = 3.355, *p* = .187) or and the Age × Language Group interaction (*β* = −0.547, *p* = .104; *χ*^2^(1) = 2.65, *p* = .104) did not predict performance on these open-ended questions.

## GENERAL DISCUSSION

We conducted a cross-linguistic study to examine how features of temporal language impact children’s learning of deictic temporal terms, like “yesterday,” focusing on German and English. German’s lexicon includes additional single words for time points like “the day before yesterday” (*vorgestern*) and “the day after tomorrow” (*übermorgen*), which could facilitate earlier acquisition of temporal terms in children learning German as compared to English. This study was the first to explore cross-linguistic differences in 3-to-7-year-old children’s learning of deictic temporal terms in both languages. A summary of our main findings is provided in the Results section (see Table 2).

Our primary result was that German-speaking children showed greater knowledge of the precise meanings for deictic temporal terms than English-speaking children. Importantly, this was true not only for the terms that are lexicalized only in German and must be expressed by whole phrases in English, e.g., “the day before yesterday”, but, critically, also for more basic temporal concepts that are expressed by single words in both languages, like “yesterday” and “tomorrow.” The advantage was driven primarily by German-speakers’ elevated understanding of temporal remoteness (distance from *now*), while comprehension of deictic status (whether a term referenced the past vs. future) was similar across groups. Below, we discuss these findings and their implications.

We found that German-speakers were more likely to have precise meanings for the terms *übermorgen* and *vorgestern* than English-speakers were to know the corresponding phrases. This may suggest that compound words indicating times are more easily acquired by children than whole phrases such as “the day before yesterday”. English-learners often struggle for years to learn the meanings of the terms *before* and *after* (e.g., Amidon & Carey, 1972; Blything & Cain, 2016, 2019; Blything et al., 2015; Cain & Nash, 2011; Clark, 1971), which may exacerbate the difficulty of comprehending phrases involving *tomorrow* and *yesterday*. Although there is less work examining German-speakers’ acquisition of prefixes like *vor-* and *über-*, one prior study suggests that children do not fully comprehend these prefixes out of context until age 6–8 (Mattes, 2019). Given that we found evidence of above-chance comprehension of terms involving these prefixes in children as young as 4 in the present study, it is possible that these young German-learners did not need to know the meaning of the prefix (e.g., *vor-*) to effectively contrast the whole term (e.g., *vorgestern*) with the other time words in their lexicons, a strategy we will discuss further below.

Secondly, and more interestingly, we found that German-speaking children were *also* more likely to have precise meanings for the proximal words *yesterday* and *tomorrow*, despite both languages sharing these single lexical items. This finding is consistent with theories suggesting that children do not learn deictic time words solely through individual word-to-event mappings, and instead acquire these meanings relationally, inferring each word’s place within the entire set of items in their time-word lexicon (see also Tillman et al., 2017). This aligns with prior research showing that children understand that time words form a common semantic domain by age 4 (Shatz et al., 2010), with sequential ordering emerging by age 5 (Tillman & Barner, 2015; Tillman et al., 2017), prior to acquisition of precise adult meanings. For German speakers, the presence of more terms (e.g., *vorgestern*) in the lexicon may facilitate this relational learning by narrowing the scope of other words like *gestern* and *morgen*. This pattern mirrors findings in color-word acquisition, where overextended meanings (e.g., calling a pink hue “red”) are refined as children acquire new alternatives (e.g., “pink”, Wagner et al., 2013).

To pinpoint the source of the German advantage, we assessed children’s understanding of three components of time meaning: *deictic status*, *sequential order*, and *remoteness*. The German advantage primarily emerged for *remoteness*. Across ages, German speakers exhibited greater comprehension of the distance from the present indicated by each temporal item. This advantage may stem from German’s more densely packed lexicon of time words. For example, *vorgestern* (exactly two days ago) offers a proximal neighbor to *gestern* (yesterday), helping German speakers avoid overextending *gestern* to more distant past events, a pattern commonly observed in English learners. Similarly, the presence of *übermorgen* may help constrain the temporal scope of *morgen*. In contrast, we found no overall language group differences in comprehension of *deictic status* or *sequential order*. German-speakers did, however, show a temporary advantage in sequential order knowledge specifically at age 3, the youngest age group we tested. This finding in 3-year-olds suggests that linguistic cues like lexical contrast and order-of-mention may help young children infer time-word order at an earlier age when all relevant terms consist of single words.

The absence of a German advantage in deictic status knowledge aligns with theories suggesting that grammatical tense—marked similarly in English and German—supports the acquisition of past versus future distinctions (e.g., Gleitman et al., 2005). Further supporting this claim, Maheshwari and Barner (2025) provide direct evidence that syntactic cues—particularly tense information—play a critical role in children’s early learning of deictic time words. Their cross-linguistic study of time word learning in Hindi and English found that Hindi-speaking children, who rely on overt tense markers, outperformed English-speaking children in identifying past and future references, even though Hindi uses the same word (*kal*) for both *yesterday* and *tomorrow*. Moreover, English-speaking children struggled when tense cues were absent, suggesting that syntactic structure is crucial for determining a word’s deictic status. Thus, while the present study did not explicitly test the role of syntactic cues, our findings are consistent with the view that children’s understanding of deictic distinctions is shaped by linguistic structure rather than solely by direct experience with events.

While our findings regarding specific age groups are preliminary in nature, visual inspection of the data (e.g., Figure 3) as well as some exploratory post-hoc comparisons reported in the Supplementary Materials provide a first look at the developmental trajectory of the German advantage. In particular, while language-group differences in knowledge of proximal terms (lexicalized in both languages) seemed to peak around the age of 4, differences in knowledge of distal terms (lexicalized only in German) seemed to persist later in development. Although our broad age range and large overall sample size (*n* = 304 children) is a strength of the current work, our exploratory analyses of the impact of language within specific age groups should be replicated in future cross-linguistic work targeting narrower age groups with larger and more well-balanced sample sizes of children within each, in order to get a more precise picture of the developmental time-course of cross-linguistic differences in temporal term learning.

We also found evidence that the specific task used to test children’s knowledge of temporal terms significantly influences when that knowledge appears to emerge. The present findings extended prior work using a timeline task to demonstrate that children’s understanding of time words can be broken down into distinct facets of meaning (deictic status, temporal remoteness, precise meaning) that can be independently assessed. To address the limitations of the continuous timeline task—where temporal remoteness varies by age and participant—we developed a novel, discontinuous calendar task. Using this task, children as young as age 3 demonstrated above-chance comprehension of temporal remoteness, contrasting with prior work which did not allow comparisons between children’s choices and random guessing, but nonetheless suggested that adult-like knowledge of remoteness emerged only after age 7 (Tillman et al., 2017)^13^.

Relative success on the Calendar vs. Timeline tasks also varied as a function of children’s age, with implications for the use of spatial representations of time in the classroom. On our assessment of deictic status knowledge, which was done using both tasks, children aged 4 and older consistently performed better with calendar template than the continuous timeline. This suggests that having discrete options for sticker placement, a familiar spatial convention in which each box represents a single day from left-to-right, and/or the additional temporal cues given by verbally labelling the boxes with the days of the week may have helped to scaffold children’s performance. In contrast, we did not observe significant task-based differences in younger children, with 3-year-olds actually performing slightly better on the continuous Timeline task, suggesting that, for children with more limited exposure to time-keeping artifacts such as diaries or calendars, these cues may have made the task more challenging. We also found that younger children who could perform above chance on spatial measures of temporal knowledge nonetheless often struggled to answer strictly verbal questions about the meanings of temporal terms and their relation to the days of the week, suggesting that a variety of testing formats can help to give a more nuanced picture of children’s early time knowledge.

Although we believe that the differences in time-word lexicons between English and German was the critical factor leading to the differences in children’s early acquisition of “tomorrow” and “yesterday” we observed, additional cross-cultural differences and/or differences between samples could have also played a role. In particular, we did not collect data on children’s socioeconomic status (SES), years of formal schooling (i.e., whether or not they attended preschool outside the home), parental education level, or general cognitive or language abilities. While we have no reason to believe these factors varied systematically between groups in a way that would account for the German advantage we observed, they should nevertheless be explored further in future studies. Suggesting that the German advantage in time-word learning was not due to overall increased familiarity with the conventional calendar system, children in the U.S. typically start preschool at an earlier age than children in Austria, potentially leading to earlier exposure to time concepts. Moreover, we found equal or better performance for English-speaking children than German-speaking children on all our verbal questions about the calendar system, including the ability to recite the days of the week without assistance. This finding is consistent with the hypothesis that the German advantage in the early acquisition of temporal terms is specific to their understanding of the remoteness of these items, is driven by the critical variation in the temporal lexicon size and does not reflect an overall group difference.

In conclusion, by leveraging cross-linguistic differences, this study highlights how a variety of linguistic cues shape children’s acquisition of abstract time concepts, offering a promising direction for understanding how language organizes temporal experience.

## ACKNOWLEDGMENTS

Thank you to Cole Dougherty and Austin Thought Lab research assistants Shaurya Aggarwal, Alexis Belmares, Tayler Fennell, Bela Gadgil, Amal Hashmey, Christina Howell, Vanessa Jones, Tayler Mancillas, Michael Price, Paige Wilson, and the Vienna Children’s Studies research team, especially Josephine Funke, Lea Halmer, Andrea Morgenbesser, Laura Stasch and Secil Burda-Özkan for their assistance with data collection, and Christina Schätz, Büsra Demir, Livia Hirzberger and Fabian Ottolin for help with data pre-processing. We also thank Liesbeth Forsthuber for lab management and the Kinderfreunde Wien for support in participant recruitment in Vienna. We also want to thank the many children and families who participated, without whom this research would not be possible.

## FUNDING INFORMATION

This work was funded by a UT Faculty Startup Grant awarded to K.T.

## AUTHOR CONTRIBUTIONS

Katherine Steele: Data curation; Formal analysis; Investigation; Visualization; Writing – original draft. Anna Bánki: Investigation; Methodology; Writing – review & editing. Gabriela Markova: Investigation; Methodology; Writing – review & editing. Stefanie Hoehl: Conceptualization; Resources; Supervision; Writing – review & editing. Katharine Tillman: Conceptualization; Methodology; Resources; Supervision; Writing – review & editing.

## DATA AVAILABILITY STATEMENT

The dataset for the current study is available in the Open Science Framework repository, at https://osf.io/4m9zt/.

## Notes

^1^ Throughout this paper we use “term” and “item” to refer both to single-word linguistic forms (e.g., “today”, “tomorrow”) and multiple-word expressions (e.g., “next week”, “the day after tomorrow”).^2^ This is not the case in all languages. For example, Inuktitut, an Inuit language spoken in Alaska and Northern Canada, contains different tenses that are used to discuss events in the near vs. far past and future (Swift, 2004).^3^ We abbreviate “the day before yesterday” and “the day after tomorrow” to *before-yesterday* and *after-tomorrow*, respectively.^4^ Due to experimenter error, preceding timelines were not covered for *n* = 79 English-speaking children. This did not impact our results. When added to the model, this factor did not predict performance on either the calendar or timeline task. For both the calendar and timeline tasks, the preceding and following timelines were covered for German-speaking children.^5^ Post hoc power analyses indicated high sensitivity (85–99% power) to detect the observed effect size for analyses involving proximal and distal terms for Ages 4, 5, 6, and 7 in both language groups except at age 3 for German-speakers (power = 16%). For comparisons with non-significant results and power ∼ 0% (e.g., Age 3 German-speakers), the observed effect size was minimal (e.g., *r* = 0.037), thus the results should be interpreted with caution given limited sensitivity to detect small effects.^6^ Post hoc power analyses indicated high sensitivity (87–99% power) to detect the observed effect size for analyses involving proximal and distal terms for both language groups. Power estimates were based on observed sample sizes and estimated effect sizes using one-sided Wilcoxon signed rank tests.^7^ Post hoc power analyses revealed low statistical power for detecting group differences (power = 26%) and non-significant differences should be interpreted cautiously.^8^ Post hoc power analyses revealed low statistical power for detecting differences from chance in several groups, with power as low as 65% or less. Thus, our analyses may be underpowered to reveal true group differences and these results should be interpreted with caution.^9^ In this analysis, “Item Type” refers to whether the target item the child was placing on the line was proximal or distal. Performance was also influenced by the item on the previous trial, which varied across items and was counterbalanced across item orders, as discussed in Methods (see Table 1). Specific contrasts tested on Deictic timeline 3, for children who received Order 1, were “tomorrow” (vs “yesterday”); “after-tomorrow” (vs. “tomorrow”); and “before-yesterday” (vs “after-tomorrow”). The contrasts for children who received Order 2 were “after-tomorrow” (vs “before-yesterday”), “tomorrow” (vs. “after-tomorrow”), and “yesterday” (vs. “tomorrow”).^10^ Post hoc power analyses indicated variable sensitivity to detect effects across with lower power at age 4 (22%), 5 (12%), 6 (9%), and 7 (3%) and moderate power at age 3 (78%). Consequently, non-significant differences should be interpreted with caution, as the analyses may have been underpowered to detect group differences.^11^ Post hoc power analyses indicated high sensitivity (82–99% power) to detect the observed effect sizes at each age for both language groups.^12^ One alternative explanation for our finding that children showed greater overall remoteness knowledge for proximal than distal terms is that, all else being equal, they may have preferred to place their stickers directly adjacent to the experimenter’s sticker representing ‘today’ (box 4). While this is possible in some cases, children were nonetheless far less likely to place stickers in boxes 3 or 5 for distal terms (29%) than for proximal terms (81%).^13^ It should be noted, however, that the calendar task used here also assessed a much smaller set of terms than the timeline task used by Tillman et al. (2017), which may have contributed to this difference in performance.

## Supplementary Material


