# Supplementary material for: Today, Tomorrow, and Overmorrow: The Acquisition of Deictic Temporal Terms in English and German
Source: Open Mind (Camb). 2025 Oct 29;9:1826–48. doi: 10.1162/OPMI.a.254 (PMC12594576; doi:10.1162/OPMI.a.254)
Supplement: Supplementary file 1 [file opmi-09-1826-s001.docx]

# Supplementary Materials

**Table S1**. Summary of cross-linguistic features, hypotheses, task mappings, and predicted outcomes

| **Language Feature** | **German vs. English** | **Measure (Task)** | **Predicted Outcome(s)** |
| --- | --- | --- | --- |
| Proximal temporal terms | English: yesterday/tomorrow  German: gestern/morgen | Precise meaning (Calendar) | If terms learned independently, no difference across languages  If terms learned relationally, possible German advantage |
| Distal temporal terms | English:  day after tomorrow/day before yesterday  German:  übermorgen/vorgestern | Precise meaning (Calendar) | German advantage due to relative simplicity of word vs. phrase |
| Lexicon size | Presence of additional lexicalized terms in German leads to larger lexicon | Order (Timeline) | If terms learned relationally, likely to observe language effect, but advantage could go in either direction:  Fewer words could lead to English advantage; Clearer lexical contrasts could lead to German advantage |
| Lexicon density | Presence of additional lexicalized terms in German leads to more tightly spaced lexicon | Remoteness (Calendar) | If terms learned relationally, German advantage in remoteness due to increased constraints provided by added terms |
| Tense | Both English and German have an inflectional past tense and non-inflectional future tense | Deictic status (Timeline and Calendar) | Similar deictic status performance across groups |

# Sample language background

Bilingual German-speaking children were proficient in Bosnian (*n* = 1), Chinese/Mandarin (*n* = 1), Czech (*n* = 1), English (*n* = 10), Greek, (*n* = 1), Hungarian (*n* = 1), Igbo (*n* = 1), Polish (*n* = 2), Portuguese (*n* = 2), Slovenian (*n* = 1), or Spanish (*n* = 3). Bilingual English-speaking children were proficient in Chinese/Mandarin (*n* = 5), Dinka (*n* = 1), Indonesian (*n* = 2), Italian (*n* = 1), Japanese (*n* = 1), Korean (*n* = 1), Spanish (*n* = 11), Farsi-Kurdish (*n* = 1), or Turkish (*n* = 1).

We also considered whether children’s monolingual status (monolingual vs. multilingual) predicted performance on each facet of meaning on the calendar task and timeline task. Monolingual status did not predict correct placement of items in the past or future or improve model fit of performance on the calendar task (*χ2* (1) = 0.036, *p* = .765) or the timeline task (*χ2* (1) = -0.057, *p* = .621). Additionally, monolingual status did not predict knowledge of remoteness or improve model fit (*χ2* (1) = 1.581, *p* = .209), and the same was true for precise knowledge (*χ2* (1) = 0.292, *p* = .589) on the calendar task. Finally, monolingual status did not predict order knowledge or improve model fit (*χ2* (1) = 1.924, *p* = .165) on the timeline task.

1. **Calendar Task**

**2.1 Precise Meaning**

**2.1.1. Item-level percent accuracy for precise meaning**

**Table S2.** Percent accuracy for Precise Meaning on the Calendar Task for each Term

| **Age** | **Language** | **before-yesterday** | **yesterday** | **tomorrow** | **after-tomorrow** |
| --- | --- | --- | --- | --- | --- |
| 3 | English  (*n* = 22) | 9.09 (29.4) | 42.86 (50.71) | 40.91 (50.32) | 9.09 (29.42) |
|  | German  (*n* = 38) | 7.89 (27.33) | 30.56 (46.72) | 37.84 (49.17) | 18.92 (39.71) |
| 4 | English  (*n* = 47) | 19.15 (39.77) | 34.04 (47.90) | 51.06 (50.53) | 17.02 (37.99) |
|  | German  (*n* = 42) | 33.33 (47.71) | 45.24 (50.38) | 66.67 (47.71) | 38.09 (49.15) |
| 5 | English  (*n* = 42) | 26.19 (44.50) | 66.67 (47.71) | 71.43 (45.72) | 50.00 (50.61) |
|  | German  (*n* = 39) | 56.41 (50.24) | 66.67 (47.76) | 79.49 (40.91) | 58.97 49.83) |
| 6 | English  (*n* = 28) | 53.57 (50.79) | 85.71 (35.63) | 89.29 (31.50) | 75.00 (44.10) |
|  | German  (*n* = 18) | 72.22 (46.09) | 88.89 (32.34) | 94.44 (23.57) | 61.11 (50.16) |
| 7 | English  (*n* = 12) | 83.33 (38.92) | 91.67 (28.87) | 100 (0.00) | 91.67 (28.87) |
|  | German  (*n* = 16) | 81.25 (40.31) | 93.75 (25.00) | 93.75 (25.00) | 87.50 (34.16) |
| Overall | English  (*n* = 151) | 31.1 (46.5) | 58.7 (49.4) | 66.2 (47.5) | 41.7 (49.5) |
|  | German  (*n* = 153) | 42.5 (49.6) | 57.6 (49.6) | 69.1 (46.4) | 46.7 (50.1) |

*Note*. Values represent mean percentage correct, with standard deviations in parentheses. Chance-level performance is 16.7%, or 1/6 on each trial.

**2.1.2 Post hoc analyses on performance for precise meaning**

To investigate the time course of the German advantage, we conducted post-hoc analyses including one-sided Wilcoxon signed-rank tests comparing children’s performance to chance (reported in the main text; see also **Table S5**) and two-sample Wilcoxon rank-sum tests comparing performance between German- and English-speaking children at each age. To aid interpretation of non-significant results, we conducted sensitivity power analyses using the *pwr* package in R (Champely, 2023) only for the two-sample comparisons, estimating the smallest effect sizes (SESOI) detectable with 80% power given our sample sizes. These values are reported in **Table S3 and S4**. Sensitivity analyses for the calendar task indicated that the minimal detectable effect sizes (*r*) for English vs. German comparisons ranged from 0.21 to 0.36 across ages 3–7, corresponding to small-to-moderate effects in most subgroups (**Table S3**). For the timeline task, minimal detectable *r* ranged from 0.15 to 0.26 across the same age range, reflecting similar interpretive patterns (**Table S4**). Non-significant findings in smaller subgroups may reflect effects below these thresholds rather than the absence of an effect.

**Table S3**. Minimal Detectable Effect Sizes (*r*) for English vs. German Comparisons by age on the Calendar Task

| **Age** | **English sample (*n*)** | **German sample (*n*)** | **SESOI (r, 80% power)** | **Interpretation** |
| --- | --- | --- | --- | --- |
| 3 | 44 | 76 | 0.26 | Small-to-moderate effects |
| 4 | 94 | 84 | 0.21 | Small-to-moderate effects |
| 5 | 84 | 78 | 0.22 | Small-to-moderate effects |
| 6 | 56 | 36 | 0.29 | Small-to-moderate effects |
| 7 | 24 | 32 | 0.36 | Moderate effects |

Note. SESOI = smallest effect size of interest (r) detectable with 80% power at α = 0.05 given the subgroup sample sizes. Values indicate the minimum effect that could be reliably detected; non-significant results in smaller subgroups may reflect effects below this threshold rather than the absence of an effect.

**Table S4**. Minimal Detectable Effect Sizes (*r*) for English vs. German Comparisons by age on the Timeline Task

| **Comparison** | **English sample (*n*)** | **German sample (*n*)** | **SESOI (r, 80% power)** | **Interpretation** |
| --- | --- | --- | --- | --- |
| 3 | 88 | 152 | 0.19 | Small effects |
| 4 | 188 | 168 | 0.15 | Small effects |
| 5 | 168 | 156 | 0.15 | Small effects |
| 6 | 112 | 72 | 0.21 | Small-to-moderate effects |
| 7 | 48 | 64 | 0.26 | Small-to-moderate effects |

Note. SESOI = smallest effect size of interest (r) detectable with 80% power at α = 0.05 given the subgroup sample sizes. Values indicate the minimum effect that could be reliably detected; non-significant results in smaller subgroups may reflect effects below this threshold rather than the absence of an effect.

Two-sample Wilcoxon rank-sum tests revealed no significant differences in performance between English- and German speakers on proximal or distal terms except at ages 4 and 5 for distal terms (see **Table S6**).

**Table S5**. Results from one-sided Wilcoxon signed-rank tests comparing children’s precise meaning knowledge on the calendar task to chance

| **Age** | **Language (*n*_trials_)** | **Item Type** | ***V*** | **Effect size (*r*)** | ***p*** |
| --- | --- | --- | --- | --- | --- |
| 3 | English (*n* = 44) | proximal | 621 | 0.222 | .033 |
|  |  | distal | 170 | -0.572 | 1.00 |
|  | German (*n* = 76) | proximal | 1525 | 0.037 | .159 |
|  |  | distal | 705 | -0.450 | > .99 |
| 4 | English (*n* = 94) | proximal | 2980 | 0.291 | .002 |
|  |  | distal | 1462 | -0.300 | < .99 |
|  | German (*n* = 84) | proximal | 2867 | 0.527 | < .001 |
|  |  | distal | 2085 | 0.146 | .082 |
| 5 | English (*n* = 84) | proximal | 3219 | 0.698 | < .001 |
|  |  | distal | 2192 | 0.198 | .03 |
|  | German (*n* = 78) | proximal | 2850 | 0.739 | < .001 |
|  |  | distal | 2520 | 0.552 | < .001 |
| 6 | English (*n* = 56) | proximal | 1568 | 0.839 | < .001 |
|  |  | distal | 1386 | 0.641 | < .001 |
|  | German (*n* = 36) | proximal | 660 | 0.856 | < .001 |
|  |  | distal | 588 | 0.668 | < .001 |
| 7 | English (*n* = 24) | proximal | 299 | 0.869 | < .001 |
|  |  | distal | 294 | 0.840 | < .001 |
|  | German (*n* = 32) | proximal | 525 | 0.863 | < .001 |
|  |  | distal | 513 | 0.823 | < .001 |

Note. Sensitivity power analyses (SESOI, 80% power, α = .05) are reported in Table S3.

**Table S6**. Results from two-sample Wilcoxon rank-sum tests comparing English- and German-speaking children’s precise meaning knowledge on the calendar task

| **Age** | **German-speakers (*n*_trials_)** | **English-speakers (*n*_trials_)** | **Item Type** | ***W*** | ***Z*** | **Effect Size (*r*)** | ***p*-value** |
| --- | --- | --- | --- | --- | --- | --- | --- |
| 3 | *n* = 76 | *n* = 44 | proximal | 1689 | 0.09 | 0.008 | .416 |
|  |  |  | distal | 1580 | -0.50 | -0.046 | .493 |
| 4 | *n* = 84 | *n* = 94 | proximal | 3419 | -1.54 | -0.116 | .075 |
|  |  |  | distal | 3252 | -2.03 | -0.152 | .008 |
| 5 | *n* = 78 | *n* = 84 | proximal | 3144 | -0.44 | -0.035 | .575 |
|  |  |  | distal | 2634 | -2.15 | -0.169 | .013 |
| 6 | *n* = 36 | *n* = 56 | proximal | 966 | -0.34 | -0.035 | .538 |
|  |  |  | distal | 984 | -0.19 | -0.020 | .82 |
| 7 | *n* = 32 | *n* = 24 | proximal | 392 | 0.13 | 0.018 | .75 |
|  |  |  | distal | 396 | 0.20 | 0.027 | .753 |

Note. Sensitivity power analyses (SESOI, 80% power, α = .05) are reported in Table S3.

When compared to adult performance, English-speaking 7-year-olds (*W* = 3968, *r* = -0.043, *p* = .046) and German-speaking 7-year-olds (*W* = 6120, *r* = 0.066, *p* = .005), were both statistically indistinguishable from adult performance. *Note*: English-speaking adults performed at ceiling on all questions on the calendar task, therefore German-speaking adults were not tested on the calendar task.

**2.2 Deictic Status**

**2.2.1 Post hoc analyses**

We conducted post-hoc analyses including one-sided Wilcoxon signed-rank tests comparing children’s performance to chance (reported in the main text; see also **Table S7**) and two-sample Wilcoxon rank-sum tests comparing performance between German- and English-speaking children at each age (**Table S8**).

**Table S7**. Results from one-sided Wilcoxon signed-rank tests comparing children’s deictic status knowledge on the calendar task to chance

| **Age** | **Language (*n*_trials_)** | **Item Type** | ***V*** | **Effect size (*r*)** | ***p*** |
| --- | --- | --- | --- | --- | --- |
| 3 | English (*n* = 44) | Proximal | 540 | 0.079 | .276 |
|  |  | Distal | 517.5 | 0.040 | .384 |
|  | German (*n* = 76) | Proximal | 1578.5 | 0.069 | .247 |
|  |  | Distal | 1501.5 | 0.023 | .410 |
| 4 | English (*n* = 94) | Proximal | 2707.5 | 0.185 | .020 |
|  |  | Distal | 3040 | 0.314 | < .001 |
|  | German (*n* = 84) | Proximal | 2465 | 0.331 | < .001 |
|  |  | Distal | 2550 | 0.372 | < .001 |
| 5 | English (*n* = 84) | Proximal | 2890 | 0.538 | < .001 |
|  |  | Distal | 2762.5 | 0.476 | < .001 |
|  | German (*n* = 78) | Proximal | 2409.5 | 0.490 | < .001 |
|  |  | Distal | 2370 | 0.468 | < .001 |
| 6 | English (*n* = 56) | Proximal | 1539 | 0.808 | < .001 |
|  |  | Distal | 1368 | 0.621 | < .001 |
|  | German (*n* = 36) | Proximal | 647.5 | 0.823 | < .001 |
|  |  | Distal | 555 | 0.581 | < .001 |
| 7 | English (*n* = 24) | Proximal | 300 | 0.875 | < .001 |
|  |  | Distal | 287.5 | 0.801 | < .001 |
|  | German (*n* = 32) | Proximal | 511.5 | 0.818 | < .001 |
|  |  | Distal | 511.5 | 0.818 | < .001 |

Note. Sensitivity power analyses (SESOI, 80% power, α = .05) are reported in Table S3.

Two-sample Wilcoxon rank-sum tests revealed no significant differences in performance on proximal or distal terms at each age with one exception: 6-year-olds performance was significantly higher on proximal trials relative to distal trials (see **Table 8**).

**Table S8**. Results from two-sample Wilcoxon rank-sum tests comparing deictic status knowledge for proximal and distal terms on the calendar task

| **Age** | **Proximal % Accuracy (*M*[*SD*])** | **Distal % Accuracy(*M*[*SD*])** | **Trials** | ***W*** | ***Z*** | **Effect Size (*r*)** | ***p*-value** |
| --- | --- | --- | --- | --- | --- | --- | --- |
| 3 | 54.2(50.0) | 51.7(50.2) | *n* = 120 | 7020 | -0.33 | -0.022 | .699 |
| 4 | 64.6(48) | 69.7(46.1) | *n* = 178 | 16643 | 0.83 | 0.044 | .311 |
| 5 | 79.6(40.4) | 77.2(42.1) | *n* = 162 | 12798 | -0.38 | -0.021 | .590 |
| 6 | 96.7(17.9) | 84.8(36.1) | *n* = 92 | 3726 | -1.40 | -0.103 | **.005** |
| 7 | 98.2(13.4) | 96.4(18.7) | *n* = 56 | 1540 | -0.16 | -0.015 | .567 |

Note. Sensitivity power analyses (SESOI, 80% power, α = .05) are reported in Table S3.

When compared to adult performance, English-speaking 7-year-olds (*W* = 4184, *r* = -0.007, *p* = .618) but not German-speaking 7-year-olds (*W* = 5744, *r* = 0.015, *p* = .290), were adult-like. English-speaking adults performed at ceiling on all questions on the calendar task, therefore German-speaking adults were not tested on the calendar task.

**2.3 Temporal Remoteness**

**2.3.1 Post hoc analyses**

We conducted post-hoc analyses including one-sided Wilcoxon signed-rank tests comparing children’s performance to chance (reported in the main text; see also **Table S9**).

**Table S9**. Results from one-sided Wilcoxon signed-rank tests comparing children’s temporal remoteness knowledge to chance

| **Age** | **Language (*n*_trials_)** | **Item Type** | ***V*** | **Effect size (*r*)** | ***p*** |
| --- | --- | --- | --- | --- | --- |
| 3 | English (*n* = 44) | Proximal | 868 | 0.656 | < .001 |
|  |  | Distal | 612 | 0.206 | .079 |
|  | German (*n* = 76) | Proximal | 2550 | 0.646 | < .001 |
|  |  | Distal | 2385 | 0.548 | < .001 |
| 4 | English (*n* = 94) | Proximal | 4000 | 0.687 | < .001 |
|  |  | Distal | 3139 | 0.353 | < .001 |
|  | German (*n* = 84) | Proximal | 3399 | 0.785 | < .001 |
|  |  | Distal | 3074 | 0.627 | < .001 |
| 5 | English (*n* = 84) | Proximal | 3479 | 0.824 | < .001 |
|  |  | Distal | 3245 | 0.710 | < .001 |
|  | German (*n* = 78) | Proximal | 3036 | 0.843 | < .001 |
|  |  | Distal | 2891 | 0.762 | < .001 |
| 6 | English (*n* = 56) | Proximal | 1595 | 0.869 | < .001 |
|  |  | Distal | 1541 | 0.810 | < .001 |
|  | German (*n* = 36) | Proximal | 666 | 0.872 | < .001 |
|  |  | Distal | 660 | 0.856 | < .001 |
| 7 | English (*n* = 24) | Proximal | 299 | 0.869 | < .001 |
|  |  | Distal | 294 | 0.840 | < .001 |
|  | German (*n* = 32) | Proximal | 525 | 0.863 | < .001 |
|  |  | Distal | 522 | 0.853 | < .001 |

Note. Sensitivity power analyses (SESOI, 80% power, α = .05) are reported in Table S3.

At age 7, English-speaking (*W* = 3920, *r* = -0.051, *p* = .006) and German-speaking (*W* = 6008, *r* = 0.053, *p* = .006) performance was significantly different from English-speaking adults.

1. **Timeline analyses**

Table 1 in the main text lists the four items tested on each of four spatial timelines.

Analyses in the main text are limited to Deictic Timeline 3, on which children placed the terms *yesterday*, *tomorrow*, *before-yesterday*, and *after-tomorrow*. Focusing on this task allowed us to directly compare performance on more proximal terms that are lexicalized in both languages (*yesterday*/*tomorrow*) vs. more distal ones that are lexicalized only in German (*before-yesterday*/*after-tomorrow*), and also to directly compare performance on the Timeline task with the and Calendar task, which tested only those four items.

Prior to this most critical timeline, children completed three others. The first tested children’s ability to map temporal events (e.g., their last birthday) to the line. This served both to familiarize children with the task and as a control measure, and we did not expect to find any language group differences in performance. As reported below, we did not observe any.

Children also completed two additional deictic timelines which tested a larger overall set of terms, including *last week, next week, tonight, this morning, last year, and next year* (see **Table 1**, main text)*.* Inclusion of this larger set allowed us to test whether the differences in the German and English temporal lexicons impacted children’s acquisition of the deictic status and sequential ordering of terms that were both even more proximal (e.g., this morning) than e.g., “yesterday”, and even more distal than e.g., “the day after tomorrow” (e.g., next week), allowing us to further assess how the critical cross-linguistic difference impacted performance.

Based on prior research indicating that acquisition of deictic status is supported by tense cues, we did not expect to see language group differences in children’s comprehension of this facet of meaning, given that both languages have similar tense structures. As reported below, this is indeed consistent with our results. On the other hand, theories in which time-words are learned relationally might predict that we would still find differences in sequential order knowledge across the larger set of temporal terms (including those that are quite distant from vorgestern/day-before-yesterday), given that all terms are acquired by virtue of their relation to the full set. Nonetheless, we did not find evidence of a language group difference in sequential order knowledge across all three deictic timelines.

**3.1 Post hoc analyses on Deictic Timeline 3**

**3.1.1 Knowledge of Deictic Status**

We conducted post-hoc analyses including one-sided Wilcoxon signed-rank tests comparing children’s performance to chance (reported in the main text; see also **Table S10**).

**Table S10**. Results from one-sided Wilcoxon signed-rank tests comparing children’s deictic status knowledge on the Deictic Timeline 3 to chance

| **Age** | **Language (*n*_trials_)** | ***V*** | **Effect size (*r*)** | ***p*** |
| --- | --- | --- | --- | --- |
| 3 | English (*n* = 88) | 1827.5 | -0.058 | .415 |
|  | German (*n* = 152) | 6750 | 0.140 | .005 |
| 4 | English (*n* = 188) | 10082.5 | 0.117 | .006 |
|  | German (*n* = 168) | 8534.5 | 0.176 | .004 |
| 5 | English (*n* = 168) | 10224.5 | 0.382 | <.001 |
|  | German (*n* = 156) | 7982.5 | 0.263 | < .001 |
| 6 | English (*n* = 112) | 5028.5 | 0.511 | <.001 |
|  | German (*n* = 72) | 1898 | 0.386 | <.001 |
| 7 | English (*n* = 48) | 1053.5 | 0.689 | <.001 |
|  | German (*n* = 64) | 1787.5 | 0.625 | <.001 |

Note. Sensitivity power analyses (SESOI, 80% power, α = .05) are reported in Table S4.

Compared to adult performance, German-speaking (*W* = 108056, *r* = 0.131, p < .001) and English-speaking 7-year-olds (*W* = 62328, *r* = -0.069, *p* < .001) did not demonstrate adult-like performance.

**3.1.2 Knowledge of Sequential Order**

We conducted post-hoc analyses including one-sided Wilcoxon signed-rank tests comparing children’s performance to chance (reported in the main text; see also **Table S11**) and two-sample Wilcoxon rank-sum tests comparing performance between German- and English-speaking children at each age (reported in the main text; see also **Table S12**).

**Table S11**. Results from one-sided Wilcoxon signed-rank tests comparing children’s sequential order knowledge on the Deictic Timeline 3 to chance

| **Age** | **Language (*n*_trials_)** | ***V*** | **Effect size (*r*)** | ***p*** |
| --- | --- | --- | --- | --- |
| 3 | English (*n* = 66) | 896 | -0.165 | .812 |
|  | German (*n* = 114) | 3920 | 0.170 | .003 |
| 4 | English (*n* = 141) | 5629.5 | 0.108 | .021 |
|  | German (*n* = 126) | 5207 | 0.262 | < .001 |
| 5 | English (*n* = 126) | 5778.5 | 0.386 | < .001 |
|  | German (*n* = 117) | 4945 | 0.376 | < .001 |
| 6 | English (*n* = 84) | 3187.5 | 0.682 | < .001 |
|  | German (*n* = 54) | 1237.5 | 0.580 | < .001 |
| 7 | English (*n* = 36) | 592 | 0.678 | < .001 |
|  | German (*n* = 48) | 1029 | 0.653 | < .001 |

Note. Sensitivity power analyses (SESOI, 80% power, α = .05) are reported in Table S4.

**Table S12**. Results from two-sample Wilcoxon rank-sum tests comparing English- and German-speaking children’s sequential order knowledge on the Deictic Timeline 3

| **Age** | **German-speakers (*n*_trials_)** | **English-speakers (*n*_trials_)** | ***W*** | **Effect size (*r*)** | ***p*** |
| --- | --- | --- | --- | --- | --- |
| 3 | *n* = 114 | *n* = 66 | 2845.5 | -0.203 | .018 |
| 4 | *n* = 126 | *n* = 141 | 8139 | -0.072 | .288 |
| 5 | *n* = 117 | *n* = 126 | 6951 | -0.049 | .573 |
| 6 | *n* = 54 | *n* = 84 | 2403 | 0.050 | .315 |
| 7 | *n* = 48 | *n* = 36 | 876 | 0.012 | .853 |

Note. Sensitivity power analyses (SESOI, 80% power, α = .05) are reported in Table S4.

**3.2 Analyses including all ‘Deictic’ Timelines**

**3.2.1 Knowledge of Deictic Status**

We modeled correct placement of deictic terms in the past vs. future across the three ‘Deictic’ timelines from Language Group (English vs. German), Age, and their two-way interaction. The model revealed a significant effect of Age (𝛽 = 0.484, *p* < .001; *χ2* (1) = 72.33, *p* < .001), but no significant effect of Language Group (𝛽 = 0.048, *p* = .361; *χ2* (1) = 0.541, *p* = .462) or interaction (𝛽 = 0.103, *p* = .0616; *χ2* (1) = 3.49, *p* =.062). Overall, performance in both language groups improved with age (see **Figure S1**), and above-chance performance (50%) emerged at age 3 (*V* = 209078, *p* < .001, *r* = 0.529).

**Figure S1**

*Deictic Status Knowledge on the Timeline Task*


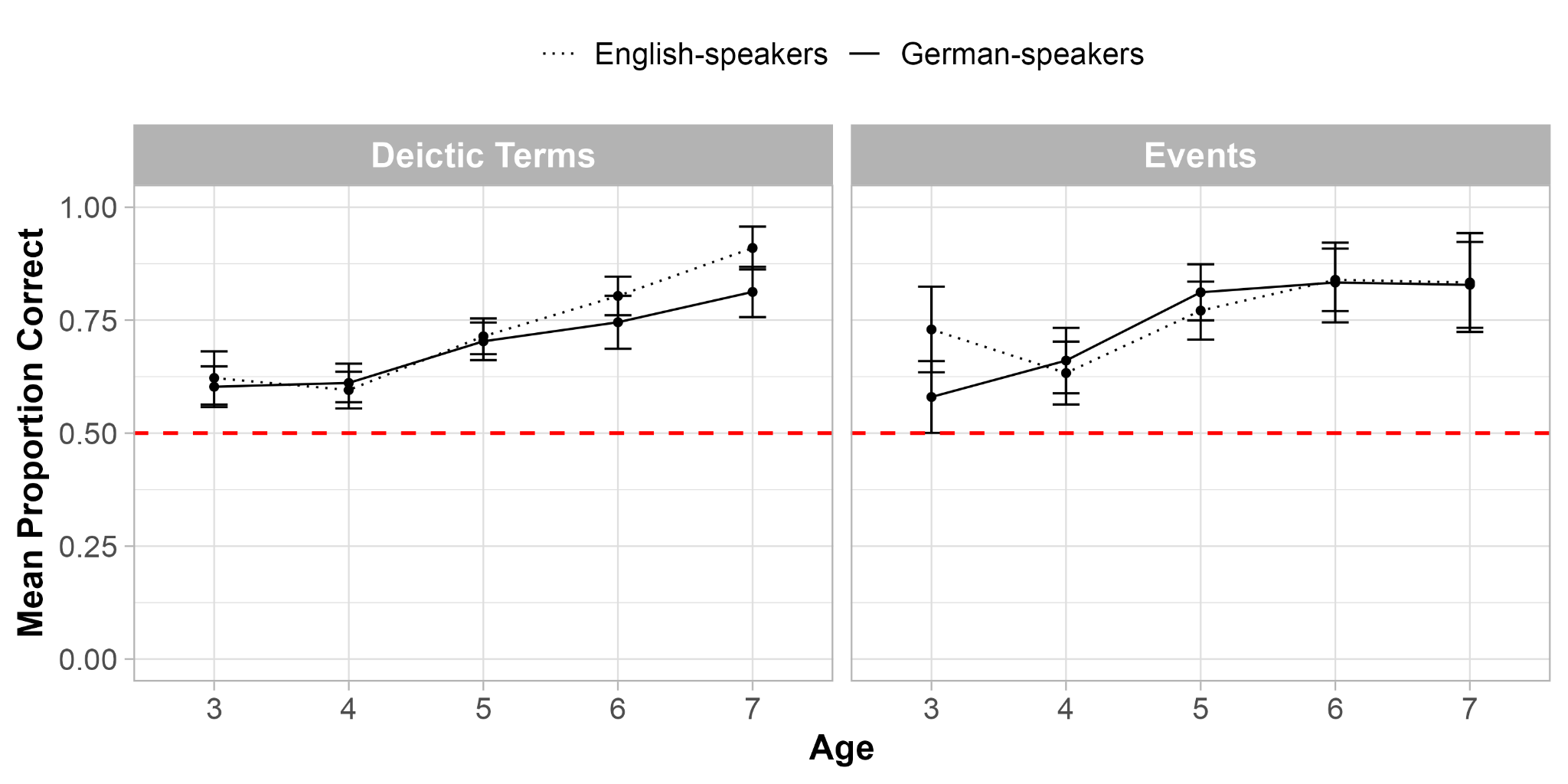


*Note*. ‘Proportion Correct’ indicates the proportion of correct placements in the past or future for events or deictic terms across three ‘Deictic’ timelines (see **Table 1** in main text) by language group (*n* = 153 German speakers, *n* = 151 English speakers) and by age (in years). Error bars represent 95% confidence intervals. The dashed red line indicates chance performance (at 50%).

**3.2.2 Knowledge of Sequential Order**

To assess children’s knowledge of the sequential ordering of deictic terms on the Timeline task, we constructed a model to predict order knowledge from Language Group (German vs. English), Age, and their two-way interaction. The model revealed a main effect of Age (𝛽 = 0.653, *p* < .001; *χ2* (1) = 95.56, *p* < .001), but no effect of Language Group (𝛽 = -0.053, *p* =.613; *χ2* (1) = 0.0824, *p* = .774) or two-way interaction between Language Group x Age (𝛽 = -0.181, *p* =.104; *χ2* (1) = 2.65, *p* = .104). As shown in **Figure S2**, mean accuracy improved monotonically with age: *M*_3_ = 0.57 < *M*_4_ = 0.61 < *M*_5_ = 0.69 < *M*_6_ = 0.79 < *M*_7_ = 0.87.

**Figure S2**

*Order Knowledge on the Timeline Task*


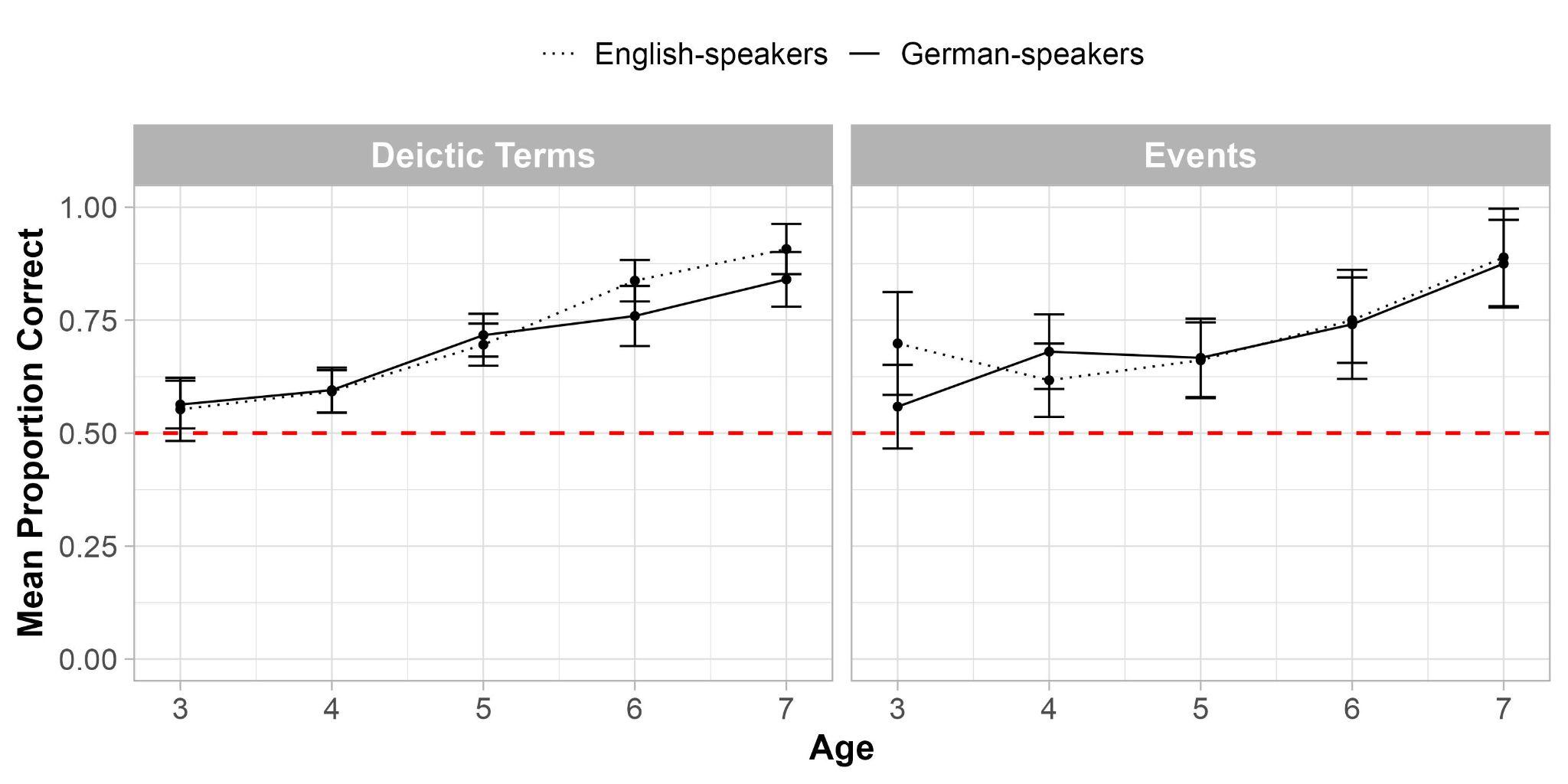


*Note*. ‘Mean Proportion Correct’ represents knowledge of sequential order by Language Group (*n* = 153 German speakers, *n* = 151 English speakers) for events and deictic terms by age (in years). We calculated ‘proportion correct’ from individuals’ average performance for event and deictic timelines separately. Error bars represent 95% confidence intervals; the dashed red line is chance performance per trial.

**3.3 Performance on the ‘Event’ Timeline**

**3.3.1 Knowledge of Deictic Status**

We constructed a model to test whether Language Group (English vs. German), Age, and/or their two-way interaction predicted correct placement of events in the past vs. future on the ‘Event’ Timeline. We found a main effect of Age (𝛽 = 0.494, *p* < .001; *χ2* (1) = 36.59, *p* < .001) but no effect of Language Group (𝛽 =-0.0002, *p* = .998) or two-way interaction (𝛽 = -0.103, *p* = .207). Overall, performance in both language groups improved with age and above-chance (50%) performance emerged at age 3 (*V* = 17582, *p* < .001, *r* = 0.187; see **Figure S1**).

**3.3.3 Knowledge of Sequential Order**

We next constructed a model to predict order accuracy for events on the timeline task from Language Group (German vs. English), Age, and/or their two-way interaction. This model revealed a main effect of Language Group (𝛽 = -0.575, *p* < .001; *χ2* (1) = 5.36, *p* = .021), Age (𝛽 = -0.509, *p* < .001; *χ2* (1) = 4.493, *p* = .034), and a two-way interaction between Language Group x Age (𝛽 = 1.318, *p* < .001; *χ2* (1) = 35.45, *p* < .001). As shown in **Figure S2**, mean accuracy improved monotonically with age: *M*_3_ = 0.61 < *M*_4_ = 0.65 < *M*_5_ = 0.66 < *M*_6_ = 0.75< *M*_7_ = 0.88.

To investigate the significant interaction between Language and Age, we conducted post hoc two-sample Wilcoxon rank-sum tests comparing German- and English-speaking children’s performance at each age. No significant differences emerged at any age (see **Table S13**).

**Table S13**. Results from two-sample Wilcoxon rank-sum tests comparing ‘event’ timeline performance in German and English

| **Age** | **German-speakers (*n*_trials_)** | **English-speakers (*n*_trials_)** | ***W*** | **Effect size (*r*)** | ***p*** |
| --- | --- | --- | --- | --- | --- |
| 3 | *n* = 14 | *n* = 66 | 3985.5 | 0.049 | .070 |
| 4 | *n* = 126 | *n* = 141 | 8056.5 | -0.080 | .286 |
| 5 | *n* = 117 | *n* = 126 | 7030 | -0.040 | .931 |
| 6 | *n* = 54 | *n* = 84 | 2289 | 0.008 | .906 |
| 7 | *n* = 48 | *n* = 36 | 876 | 0.012 | .835 |

Note. Sensitivity power analyses (SESOI, 80% power, α = .05) are reported in Table S4.
